# Glucocorticoid-centered drug spectrum of non-jaw osteonecrosis, a FAERS pharmacovigilance study with independent validation in Canada Vigilance and JADER

**DOI:** 10.3389/fphar.2026.1977191

**Published:** 2026-10-21

**Authors:** Lisheng Qi, Yu Qiu, Xiaobin Wang, Tao Zhang, Huacai Jiang

**Affiliations:** 1 Department of Spine Surgery, The Affiliated Traditional Chinese Medicine Hospital, Southwest Medical University, Luzhou, Sichuan, China; 2 Department of Joint Orthopedics, The First People’s Hospital of Yibin, Yibin, Sichuan, China; 3 Department of Joint Orthopedics, The Affiliated Traditional Chinese Medicine Hospital, Southwest Medical University, Luzhou, Sichuan, China

**Keywords:** disproportionality analysis, Food and Drug Administration Adverse Event Reporting System, glucocorticoids, jaw-propensity index, non-jaw osteonecrosis, pharmacovigilance

## Abstract

**Objective:**

To distinguish jaw misclassification and litigation-related reporting from non-jaw osteonecrosis signals and define their reproducible spectrum.

**Methods:**

We analyzed 20,690,895 deduplicated Food and Drug Administration Adverse Event Reporting System (FAERS) reports (2004Q1–2026Q2), using report-level adjudication and four disproportionality criteria. A descriptive jaw-propensity index (JPI) measured the share of each drug’s osteonecrosis reports coded as “jaw.” Reporter restrictions and comparisons with Canada Vigilance and the Japanese Adverse Drug Event Report (JADER) database examined reporting bias. These external databases recorded less solicitation. Additional analyses included glucocorticoid stratification, empirical null calibration with unmasking, indication restriction of every surviving non-glucocorticoid drug, time-to-onset modeling, and machine learning. Estimates are reported with 95% confidence intervals.

**Results:**

Of 47 signals, 27 passed jaw exclusion (JPI < 0.50) and reporter restriction; the final core was unchanged across thresholds of 0.30–0.70. Tenofovir disoproxil’s reporting odds ratio (ROR) fell from 157.9 (153.4–162.6) to 6.33 (5.38–7.45) among health professionals; 84.5% of cases were lawyer-sourced. Canada Vigilance (6.24, 2.00–19.4) and JADER (10.5, 6.60–16.8) were consistent with a litigation-inflated FAERS estimate. Glucocorticoids were replicated in both databases (class RORs 10.3, 54.4). Drug JPI rankings were concordant (r = 0.985, 0.960). All 34 negative controls had ROR < 1 (median 0.16); unmasking rescaled other estimates approximately 2.35-fold. Within-indication analyses detected no residual excess for mycophenolate (0.89) or voxelotor (0.97). Asparaginase combined a within-leukemia residual signal (7.14, 5.10–10.0) with mechanistic evidence and JADER replication (75.7).

**Conclusion:**

The reproducible spectrum is glucocorticoid-centered, with the asparaginase class the best-supported residual and three drugs unresolved. Jaw misclassification, reporting context, and indication explain much of the apparent spectrum. Spontaneous reports cannot establish causation; these adjudications require cohort confirmation. The JPI and cross-jurisdiction comparisons may inform analyses of other misclassification-prone endpoints.

## 1 Introduction

Osteonecrosis of the femoral head (ONFH) and other extra-oral sites is a disabling complication of glucocorticoid therapy, alcohol abuse, trauma, coagulopathy, sickle-cell disease, and Gaucher disease (Mankin and Mankin, 1992; Mont and Hungerford, 1995; Fukushima et al., 2010; Milner et al., 1991; Wenstrup et al., 2002). The condition arises when the blood supply to bone is interrupted, leading to osteocyte and marrow death, subchondral fracture, and eventual collapse of the articular surface (Assouline-Dayan et al., 2002; Zhou et al., 2026). Throughout this article, non-jaw osteonecrosis denotes osteonecrosis at any extra-oral site. Avascular necrosis is used as a clinical synonym for osteonecrosis, whereas ONFH refers specifically to involvement of the femoral head. The femoral head is the most common site, although the humeral head, femoral condyles, and talus are also affected.

The causal drug spectrum is conventionally reported as glucocorticoids, bisphosphonates, and antiretrovirals, a list that conflates three pharmacologically and temporally distinct phenomena. Glucocorticoid osteonecrosis is a subacute, dose-related complication of weeks-to-months exposure (Weinstein, 2012; Powell et al., 2010; Koo et al., 2002; Kerachian et al., 2009). Bisphosphonate osteonecrosis is, in almost all cases, osteonecrosis *of the jaw* (ONJ), a delayed cumulative-toxicity entity with a different anatomy, mechanism, and natural history (Ruggiero et al., 2014; Khosla et al., 2007; Ruggiero and Mehrotra, 2009; Ruggiero et al., 2022). Antiretroviral osteonecrosis reporting appears in FAERS with implausibly high reporting odds ratios (ROR > 100) that are characteristic of mass tort solicitation rather than pharmacology (Miller et al., 2002; Glesby et al., 2001; Morse et al., 2007; Grant and Cotter, 2016; Grigsby et al., 2010).

Two structural features of spontaneous-reporting data have prevented a clean answer. First, the vocabulary collapses the endpoint; MedDRA has no AVASCULAR NECROSIS preferred term (Brown et al., 1999), so extra-oral osteonecrosis is reported under the site-unspecified OSTEONECROSIS term, which is numerically dominated by jaw ONJ and by the drugs that cause it. A preferred-term-level analysis therefore cannot separate the two, and any study that treats OSTEONECROSIS as a single endpoint inherits the bisphosphonate–ONJ signal, as recent term-level analyses of FAERS illustrate (Zhong et al., 2025). Second, litigation recruits reports, and lawyer-submitted and litigation-associated reports are known to distort disproportionality measures (Rogers et al., 2019; Muñoz and Dal Pan, 2019; Hauben and Harpaz, 2026). Osteonecrosis has been the subject of product-liability litigation against the bisphosphonates, whose jaw osteonecrosis produced a documented notoriety effect on reporting (Pariente et al., 2007; de Boissieu et al., 2014), and more recently of a mass tort over tenofovir disoproxil fumarate for bone and renal injury (Supreme Court of California, 2026). As a result, litigation-sourced reports inflate specific signals in a way that is independent of biology.

The present study aims to build a report-level, full-drug-spectrum scan of non-jaw osteonecrosis that corrects both biases simultaneously and validates the result externally. Three hypotheses structure the work. (H1) Report-level adjudication and a descriptive per-drug jaw-propensity index are sufficient to separate jaw-contaminated from genuine signals; (H2) after accounting for glucocorticoid co-exposure, most signals collapse into the glucocorticoid stratum, leaving few mechanistically specific residuals; and (H3) the Weibull shape parameter of time-to-onset discriminates the subacute glucocorticoid mechanism from the delayed antiresorptive mechanism.

## 2 Materials and methods

### 2.1 Data source and collection

Data from the first quarter of 2004 to the second quarter of 2026 (90 quarterly releases) were obtained from the FAERS database (U.S. Food and Drug Administration, 2026). Each quarterly release consists of seven datasets, namely DEMO (demographic and administrative information), REAC (MedDRA-coded adverse events), DRUG (reported drugs and biological products), OUTC (patient outcomes), RPSR (report sources), THER (drug therapy start and end dates), and INDI (MedDRA-coded indications). Duplicate reports were removed in accordance with U.S. Food and Drug Administration (FDA) recommendations using a three-stage cascade on CASEID, FDA_DT, and PRIMARYID, whereby the report with the most recent FDA_DT was retained for records with the same CASEID, and if both were identical, the record with the larger PRIMARYID was kept. This removed 4,121,529 duplicate rows, leaving 20,690,895 unique reports.

### 2.2 Case definition (report-level adjudication)

Because FAERS lacks an AVASCULAR NECROSIS preferred term, non-jaw osteonecrosis was defined at the report level using a curated preferred term (PT) vocabulary. Non-jaw explicit terms (five PTs) comprised ASEPTIC NECROSIS BONE, BONE INFARCTION, BONE MARROW NECROSIS, BONE MARROW ISCHAEMIA, and PERTHES DISEASE; a single site-unspecified term (OSTEONECROSIS) was retained; and nine jaw-related terms (OSTEONECROSIS OF JAW, OSTEORADIONECROSIS, etc.) were excluded. Three schemes assigned case status. The conservative scheme (Scheme A) required an explicit non-jaw PT; the permissive scheme (Scheme B) added site-unspecified OSTEONECROSIS; and the adjudicated scheme (Scheme C, primary) subtracted from Scheme B any site-unspecified report meeting any of three jaw-suspicion rules (R1, a co-reported oral/dental/jaw preferred term; R2, an indication naming a head-and-neck or oral malignancy or a dental procedure; and R3, an antiresorptive as the suspect drug together with any co-reported oral event), which we treat as jaw ONJ mislabeled as osteonecrosis. The three rules are applied as a union. R1 accounts for 9,740 of the 9,804 exclusions, R2 adds 64 reports that R1 had not already removed, and R3, which by construction requires the same oral co-event as R1, adds none; R3 is retained as a stated part of the rule set rather than as an independent filter. Oral site terms were matched as whole words so that anatomically unrelated terms (e.g., FEMORAL, TEMPORAL) could not trigger the rules. A protopathic-bias screen then removed reports whose indication was itself osteonecrosis or its treatment (drugs given for the outcome), excluding 73 reports (0.30%) (Table 1; Figure 1).

**TABLE 1:** Report flow and case definition.

Stage	Criterion	n
Raw report rows (90 quarters, 2004Q1–2026Q2)	n.a.	24,812,424
Unique reports after 3-stage deduplication	CASEID → FDA_DT → PRIMARYID	20,690,895
Duplicate rows removed	n.a.	4,121,529
Systemic glucocorticoid co-exposed	Any role, non-local route	1,459,622 (7.1%)
Scheme A (conservative)	Explicit non-jaw PT (5 PTs)	1,521
Scheme B (permissive)	A + site-unspecified OSTEONECROSIS	33,964
Scheme C (adjudicated)	B − oral co-event/indication	24,160
Protopathic screen	Drug given *for* osteonecrosis	−73 (0.30%)
Final cases (Scheme C, final)	n.a.	24,087

**FIGURE 1:**
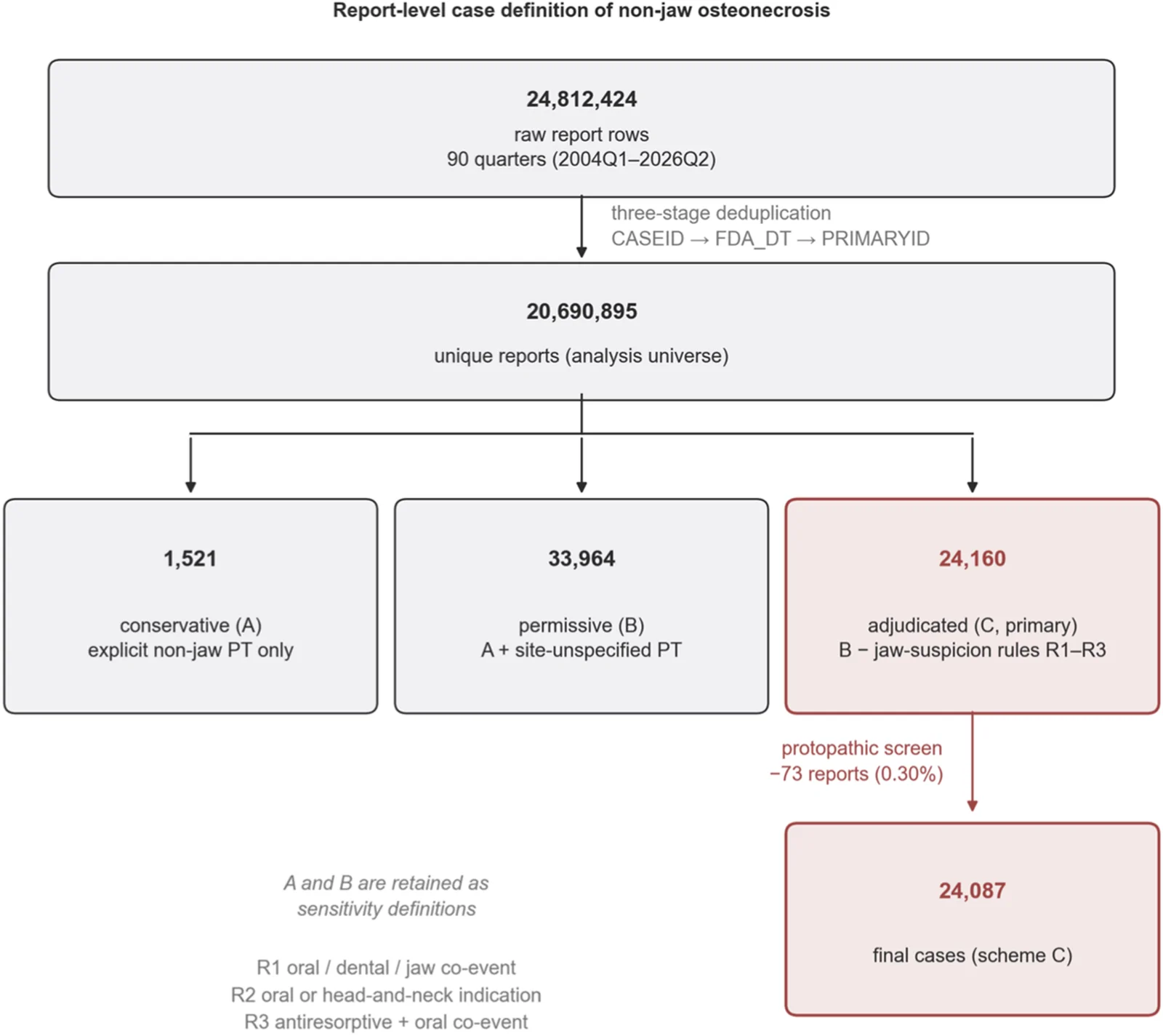
Case definition and adjudication flow. Three-scheme report-level adjudication of non-jaw osteonecrosis in FAERS (2004Q1–2026Q2). The conservative scheme **(A)** requires an explicit non-jaw preferred term; the permissive scheme **(B)** adds the site-unspecified OSTEONECROSIS term; the adjudicated scheme (C, primary) subtracts site-unspecified reports meeting any jaw-suspicion rule (R1, oral/dental/jaw co-event; R2, oral or head-and-neck indication; R3, antiresorptive suspect drug with an oral co-event) and then applies a protopathic screen (drug given for osteonecrosis). Numbers are deduplicated reports. FAERS = U.S. Food and Drug Administration (FDA) Adverse Event Reporting System; MedDRA = Medical Dictionary for Regulatory Activities; PT = preferred term. CASEID, FDA_DT, and PRIMARYID denote the case identifier, FDA receipt date, and unique report identifier, respectively.

### 2.3 Drug mapping to ingredients

Suspect drugs (role_cod = PS) were normalized and mapped to RxNorm ingredients via the RxNav API (search → approximate term → tty = IN) (Nelson et al., 2011), with two audited fallbacks, namely substring matching against the recovered ingredient vocabulary, and a hand-curated brand table (ZOMETA → zoledronic acid, AREDIA → pamidronate, ELSPAR → asparaginase, etc.). Combination brands and misresolved approximate matches were corrected by pattern-based curated overrides applied to the full name inventory (FOSAVANCE → alendronate + cholecalciferol; SERETIDE → fluticasone + salmeterol; INTRON A → interferon alfa-2b), so fixed-dose combinations accumulate into their component ingredients rather than surviving as private brand-name rows. This resolved 97.7% of report volume.

### 2.4 Disproportionality analysis

For every suspect ingredient, a 2 × 2 contingency table was constructed against the analysis universe, and four signal metrics were computed, namely the reporting odds ratio (ROR) (Rothman et al., 2004), the proportional reporting ratio (PRR) with χ^2^ (Evans et al., 2001), the multi-item gamma-Poisson shrinker (MGPS; EBGM05, implemented in the one-component gamma-Poisson form specified in Supplementary Table S1) (DuMouchel, 1999), and the Bayesian confidence propagation neural network (BCPNN; IC025) (Bate et al., 1998). A drug was considered a robust signal only if all four criteria were met simultaneously (Supplementary Table S1). The four measures have complementary weaknesses, in that ROR and PRR are frequentist ratios that become unstable when the case count is small, whereas EBGM05 and IC025 shrink small counts toward the null and are conservative for rarely reported drugs (van Puijenbroek et al., 2002; Hauben and Zhou, 2003; Bate and Evans, 2009; Almenoff et al., 2007; Norén et al., 2006; Norén et al., 2013; Sakaeda et al., 2013). Requiring all four therefore serves as a consensus filter that deliberately trades sensitivity for specificity. In a scan of 432 ingredients, where false-positive signals are the dominant risk, this is the appropriate direction of the trade-off. The scan was a single pooled analysis of the 90 quarters, not a quarter-by-quarter search. The Fisher exact test with Bonferroni correction across the 432 ingredients tested (a ≥ 3) was applied to each 2 × 2 table.

### 2.5 Jaw-propensity index (JPI)

For each drug, the jaw-propensity index (JPI) was computed as JPI = N_jaw/N_total, where N_total counts the distinct reports in the union of the jaw-term set and the non-jaw case set, so a report carrying both terms is counted once. This is the fraction of its osteonecrosis-family reports carrying the explicit OSTEONECROSIS OF JAW term. The JPI is a descriptive contamination index rather than a validated diagnostic instrument because it is computed from the same jaw-site preferred term that defines clinical jaw-centeredness. Therefore, any comparison with drugs already known to cause jaw osteonecrosis is a consistency check rather than an independent validation, and we present it as such. A JPI value near 1 marks a drug whose osteonecrosis reports are almost all coded to the jaw term, and a JPI near 0 marks a drug whose reports rarely are. The index describes how the reports were coded; it cannot establish the anatomical site of a report that does not name one. Drugs with JPI ≥ 0.50 were classified as high jaw propensity and treated as contaminated for the non-jaw headline. The 0.50 cut is a pre-specified majority rule; a drug is called “jaw-dominated” when more than half of its osteonecrosis-family reports are explicitly jaw, and this does not depend on the results. Its influence was examined in two ways. First, the threshold was swept from 0.30 to 0.70 in steps of 0.05, and the surviving-signal set was rebuilt at each value, recording in both directions which drugs would leave the set and which would enter it (Supplementary Table S2; Supplementary Figure S1). Second, the classification was compared against a knowledge-based reference set (six drugs whose osteonecrosis is established as being jaw-centered, the five bisphosphonates plus denosumab, versus the systemic glucocorticoids of the signal set whose osteonecrosis is established as being extra-oral), computing sensitivity, specificity, and the Youden J index, and the JPI of the reference drugs was recomputed in the two external databases.

### 2.6 Glucocorticoid co-exposure stratification (H2)

A report was flagged as “glucocorticoid-exposed” (GC-exposed) if any drug entry matched a systemic glucocorticoid regular expression (generics, brands and class terms) and was not a local route or androgen product. For each robust signal, the stratified ROR in the GC-exposed and GC-unexposed strata was computed, together with the additive interaction (RERI) (Rothman et al., 2008), the Ω shrinkage measure of drug × GC interaction (Norén et al., 2008), and a Firth-penalized logistic regression (Firth, 1993) adjusted for age (an indicator for age ≥ 60 and a second for age not recorded), male sex (reports without a recorded sex falling in the reference category), report year as a centered linear term, and glucocorticoid co-exposure, fitted for the 15 largest signals one drug at a time to avoid collinearity between fixed-dose-combination components. A pre-specified evaluability rule was locked before inspection, with a GC-negative (GC−) ≥ 10 leading to a reported stratified ROR, 3 ≤ GC− < 10 leading to interaction measures only (reported as interaction-only, i.o., with the direction of the stratum estimate but no point estimate), and GC− < 3 leading to not evaluable.

### 2.7 Misclassification bounding

Because concomitant medication is recorded incompletely, a GC-negative report may simply be one whose glucocorticoid was never entered. Recording density (drugs per report) was measured, and for each drug with a reportable GC-unexposed stratum, an adversarial tipping fraction was computed. Let a, b, c, and d be the cells of the drug’s 2 × 2 table inside the GC-unexposed stratum. Under-recording moves truly GC-exposed cases into that stratum, so the adversarial model assumes that all under-recording is concentrated where it damages the GC-independence call most, in the drug-exposed case cell a. A fraction f of the drug’s GC-negative cases is reassigned to the GC-exposed stratum (a shrinks to (1 − f); the other cells are unchanged). f is increased from 0 in steps of 0.01, and the tipping fraction f* is the smallest f at which the lower 95% bound of the GC-negative ROR reaches 1. Drugs whose lower bound is still above 1 at f = 0.99 are reported as greater than 0.99. Calls were graded as fragile (f* < 0.30), moderate (0.30–0.59), or robust (≥ 0.60), and f* was compared with the observed recording density. As a worked example, mycophenolate has 43 GC-negative cases with a GC-negative ROR of 1.52 (lower bound 1.13); reassigning 10% of them, approximately 5 reports, is enough to bring the lower bound to 1, whereas imiglucerase (117 GC-negative cases) would require 94%, or approximately 110 reports. All tipping fractions are listed in Supplementary Table S3 and Supplementary Figure S2.

### 2.8 Empirical null calibration

Thirty-four negative controls (antihistamines, proton-pump inhibitors, antihypertensives and diuretics, oral antidiabetics, statins, selective serotonin reuptake inhibitors, levothyroxine, acetaminophen, gabapentinoids, salbutamol (albuterol in the FAERS vocabulary), non-steroidal anti-inflammatory drugs, and montelukast, none with a plausible osteonecrosis mechanism; anticoagulants, and vitamin D excluded *a priori*) were selected (Supplementary Table S4). The observed log-ROR distribution of these controls was fitted to a normal null N (μ, σ) on the natural-log scale (Schuemie et al., 2014), yielding the systematic-error model against which the *p*-value and 95% confidence interval of every signal were recalibrated. This also quantifies the competitive masking that depressed all non-headline RORs (Maignen et al., 2014; Wang et al., 2010; Hauben and Maignen, 2017). Masking was then made explicit with an unmasking analysis, in which the primary scan was recomputed after removing from the analysis universe every report in which a masking-cluster drug was a suspect drug, in two pre-specified stages. First, the six antiresorptives and the six litigated antiretrovirals (the two artifactual clusters) were removed, and then additionally the seven drugs of the systemic glucocorticoid class, which includes betamethasone and is one drug larger than the six-drug glucocorticoid core of the signal set, were removed. At each stage, the negative-control distribution, the ROR and four-criterion status of every primary signal, and any ingredient newly meeting all four criteria were recorded (Supplementary Table S5; Supplementary Figure S3).

### 2.9 Time-to-onset (H3)

Time-to-onset was computed report-by-report as EVENT_DT − THER.START_DT, joined through the drug sequence number so that onset is attributed to a specific drug entry. The entry may hold the primary or the secondary suspect role because the onset date is recorded per drug entry, and restricting attribution to the primary suspect alone, with the same two steps applied to those entries, would leave 5,407 cases with an attributable onset (22.4%) instead of 6,532 (27.1%). One case reported through several channels reaches FAERS as several case numbers, which the deduplication cascade on CASEID cannot collapse, so onset records of the same drug that match on therapy start date, event date, sex, and country were treated as reports of one case and counted once. This removed 1,215 of 12,392 attributable onset records across the corpus. The match is evidence of duplicate reporting rather than proof of it, and it can neither recognize a duplicate that differs in one of those fields nor separate two reports that agree in all of them. A two-parameter Weibull distribution was then fitted to the onset times of each drug left with at least five such records. Onset is recorded to the day, so each record was fitted as the 1-day interval it stands for rather than as an exact time, because a same-day record entered as an exact zero lies at the origin, where the Weibull density is unbounded for α < 1, and the likelihood at the fitted parameters is not finite. The median reported for each drug is the median of that fitted distribution, not of the observed onsets, which are given alongside it in the supplement. The shape parameter α separates decreasing (α < 1), constant (α ≈ 1), and increasing (α > 1) hazard (Sauzet et al., 2013), and its 95% CI was taken from the 2.5th and 97.5th percentiles of 2,000 bootstrap resamples. A resample in which every record falls in the same 1-day interval has no maximum because the probability mass that interval carries can be driven arbitrarily close to 1 by letting α grow, so such a resample was recorded at infinity rather than at the value the optimizer returns. Where that interval is the one at the origin, the profile is instead flat in α, so the resample carries no direction and was counted without a value. A drug whose 97.5th percentile falls among resamples recorded at infinity is reported as not identified and left unclassified. A fit whose median falls below 1 day is degenerate and is tabulated but not plotted.

### 2.10 Reporting-bias sensitivity

FAERS records the occupation of the primary reporter in the field occp_cod, coded as physician (MD), pharmacist (PH), other health professional (OT), health professional (HP), lawyer (LW), or consumer (CN). Litigation-sourced reports cannot be identified with certainty, because reports solicited through law firms may be filed under the lawyer code but also reach FAERS through consumers or manufacturers with the occupation field left blank, so the lawyer code identifies only a lower bound of litigation-related reporting (Rogers et al., 2019; Muñoz and Dal Pan, 2019). No report was therefore deleted from the primary scan. Instead, the scan was recomputed under four pre-specified reporter restrictions, namely exclusion of lawyer-sourced reports (occp_cod = LW), restriction to health professional reporters (MD, PH, OT, HP), exclusion of consumer reports, and exclusion of fatal outcomes, and the lawyer-sourced share of each signal’s cases was tabulated. The health professional restriction was designated the primary litigation screen because it does not depend on the completeness of the lawyer code. A signal passed the screen if it still met all four criteria in reports from health professionals and its health-professional ROR retained at least half of the all-reporter ROR (ratio ≥ 0.5). A signal that fails this rule is carried mainly by non-professional reporting, the signature of solicited or notoriety-driven reporting (Pariente et al., 2007; Hoffman et al., 2014). The 95% CI of every restricted estimate was recorded.

### 2.11 External validation (Canada Vigilance and JADER)

Two independent regulatory databases were re-analyzed with the outcome vocabulary of the adjudicated scheme expressed in each database’s own coding system, a database-specific oral co-event exclusion standing for rule R1, and the same disproportionality metrics, with jaw involvement identified through the explicit jaw-site preferred term of each database (OSTEONECROSIS OF JAW; 顎骨壊死 in JADER). The Canada Vigilance bulk extract (release of 30 November 2024; 1,154,017 reports) (Health Canada, 2024) was parsed programmatically from its relational tables (reports, reactions, report_drug), retaining suspect-role drugs, and the reporter type field of every report was tabulated. Canadian proceedings over tenofovir disoproxil do exist (I. F. V. Gilead Sciences and Inc., 2026). What differs between the jurisdictions is how much lawyer-coded reporting of this endpoint each database received. The lawyer reporter type appears on none of the 496 Canadian osteonecrosis cases of any drug, including the three tenofovir cases among them, and on two of the 1,127 Canadian tenofovir reports of any outcome, against 5,937 of the 7,027 American tenofovir osteonecrosis cases (84.5%). Three Canadian cases cannot carry a rate of their own, so the comparison is between a corpus whose osteonecrosis cases never carry the lawyer code and one in which most tenofovir cases do. The lawyer code is a lower bound on solicitation because solicited reports also enter through the consumer channel with the occupation field blank. Therefore, this contrast measures lawyer-coded reporting rather than total recruitment, and the test compares databases on that measurement rather than on the presence or absence of a lawsuit. The Japanese Adverse Drug Event Report database (JADER, June 2026 release, 1,037,161 cases, reports carrying a reporting-quarter label from Japanese fiscal-year 2004 quarter 1 to fiscal-year 2025 quarter 4, that is to March 2026) (Pharmaceuticals and Medical Devices Agency, 2026) was parsed from its four constituent tables (patient demographics, drug, reaction and underlying disease), restricting drug exposure to the suspect role (被疑薬) and glucocorticoids to systemic non-local routes. Japanese generic names were mapped to the FAERS ingredients by substring matching, and the outcome vocabulary was expressed in MedDRA/J rather than reduced to a single term, so that 骨壊死 (osteonecrosis, site unspecified) was adjudicated by the same oral co-event exclusion (rule R1), the Japanese equivalents of the explicit non-jaw terms were added, and 顎骨壊死 (osteonecrosis of jaw) served as the jaw term, giving 1,362 adjudicated Japanese cases (1,310 site-unspecified, of which 6 were removed for an oral co-event, plus 60 carrying an explicit non-jaw term, of which 2 also carry the site-unspecified term and are counted once). The jaw-propensity index is therefore recomputed against an independent naming system under the same vocabulary and the same oral co-event exclusion. The external definitions are database-specific approximations of Scheme C rather than the identical rule. Rule R2 needs an ingredient-mapped indication table that neither external extract carries, so it is FAERS-only; rule R3 by construction requires the same oral co-event as R1 and removes nothing beyond it in any database. Both external definitions are therefore the Scheme B vocabulary with rule R1 applied. Within FAERS, the difference is small, with R2 removing 64 reports beyond R1, and R3 removing none, but the corresponding number abroad cannot be measured because neither extract carries the indication table R2 needs. In JADER, the oral exclusion is a finite list of MedDRA/J terms rather than the regular expression used in FAERS, and four of the retained Japanese cases carry an oral preferred term outside that list; excluding them would leave 1,358 cases instead of 1,362. They are retained so that the reported estimate matches the rule as stated, and the difference is disclosed here rather than corrected silently. The external comparators are therefore more permissive than the FAERS one by an amount these data cannot quantify. Whether the additional cases increase or decrease an external estimate depends on whether they fall in the exposed group or in the comparator, which these data cannot determine, so the difference is a source of uncertainty rather than a bias of known direction. Because JADER records the underlying disease of each case, the indication restriction described below was also applied to the Japanese antiretroviral estimates. The three databases differ in period, size, coding system, reporting channels, and litigation context. These differences, and the concordance observed, are tabulated in Supplementary Table S6. Concordance was assessed on direction and four-criterion status, not on equality of effect size, and only drugs with at least three external cases were considered testable.

### 2.12 Machine learning corroboration

As a cross-paradigm consistency check on the same reports, a gradient-boosted decision-tree classifier (XGBoost) (Chen and Guestrin, 2016) was trained to predict non-jaw osteonecrosis case status from report-level demographic and reporting covariates, namely age (median-imputed, with a missingness indicator), sex, reporter occupation, and systemic glucocorticoid co-exposure, in a year-stratified case-control design with five controls per case. Discrimination was summarized by the area under the receiver-operating characteristic curve (AUROC) and precision–recall area (PR-AUC), with class prevalence as the PR baseline. Feature contributions were attributed using SHapley Additive exPlanations (SHAP) (Lundberg and Lee, 2017). No disproportionality output (ROR, JPI, α) was supplied to the learner, so the ranking does not restate the primary analysis. It is computed on the same reports, however, and is therefore a check of internal consistency across two modeling paradigms rather than validation in an independent sample.

### 2.13 Indication-restricted analysis

For every non-glucocorticoid drug of the intermediate survivor set, and for tenofovir disoproxil, zidovudine, nevirapine, and taliglucerase alfa, which are retained for continuity with the submitted analysis although they are not in that set, a home indication was defined by MedDRA Preferred Term substrings in the INDI table (transplant/immunosuppression for mycophenolate; acute lymphoblastic leukemia for asparaginase and pegaspargase; HIV infection for the antiretrovirals; Gaucher disease for imiglucerase, velaglucerase alfa, taliglucerase alfa, and eliglustat; sickle-cell disease for voxelotor; hypogonadism for testosterone; hepatitis B for adefovir; chronic viral hepatitis for interferon alfa-2b; neuromyelitis optica spectrum disorder for satralizumab; acute promyelocytic leukemia for arsenic trioxide; osteoporosis or vitamin D deficiency for cholecalciferol). Two quantities were computed: the ROR of the indication itself for osteonecrosis (its confounding potential), and the ROR of the drug recomputed within reports carrying that indication, holding the disease baseline constant. The adjudication rule was fixed in advance, so that a within-indication ROR whose lower 95% bound exceeds 1 marks a residual within-indication signal (a candidate drug effect beyond the disease baseline). A lower bound at or below 1 marks an indication signal, meaning that no residual excess is detected once the disease is held constant, which is compatible with the association being carried by the disease but does not exclude a drug contribution the data are too sparse to resolve. A drug with fewer than three within-indication cases or a zero cell is not evaluable. This step, applied after jaw exclusion, reporter restriction, glucocorticoid stratification, and external replication, yields the final adjudication of every intermediate set drug reported in Table 2.

**TABLE 2:** Intermediate signal set after jaw exclusion and reporter restriction (n = 27), with the final adjudication of each drug.

Ingredient	a	ROR	JPI	HCP ROR	GC− ROR	α	Final adjudication
Prednisone	392	8.11	0.157	6.81	n.e.	0.74	Glucocorticoid core
Methylprednisolone	377	12.42	0.058	9.81	i.o.	0.54	Glucocorticoid core
Prednisolone	336	7.99	0.276	8.32	n.e.	0.56	Glucocorticoid core
Dexamethasone	312	5.52	0.235	5.42	i.o.	0.63	Glucocorticoid core
Mycophenolate mofetil	159	2.58	0.117	2.44	1.52	0.83	Indication signal (transplant; 0.89)
Voxelotor	128	5.06	0.000	23.21	5.83	1.43	Indication signal (SCD; 0.97)
Testosterone	126	2.75	0.038	8.94	3.21	n.i.	Residual within-indication signal (hypogonadism; 4.60)
Imiglucerase	118	23.57	0.017	28.16	28.43	0.95	Residual within-indication signal (Gaucher; 1.72)
Lamivudine	102	3.47	0.019	3.01	3.99	0.99	Indication signal (HIV; 0.07)
Cholecalciferol (vitamin D3)†	71	22.98	0.466	24.22	36.25	0.62	Co-prescription marker†
Triamcinolone	59	2.99	0.000	4.71	5.81	n.i.	Glucocorticoid core
Hydrocortisone	51	4.33	0.038	5.06	n.e.	0.44	Glucocorticoid core
Abacavir	46	3.76	0.042	4.44	4.33	1.11	Indication signal (HIV; 0.10)
Dolutegravir	45	2.68	0.000	3.88	2.86	0.88	Indication signal (HIV; 0.06)
Asparaginase	43	13.33	0.000	15.98	i.o.	n.i.	Residual within-indication signal (ALL; 7.14)
Velaglucerase alfa	36	16.07	0.000	16.93	19.00	0.75	Indication signal (Gaucher; 0.85)
Atazanavir	33	6.94	0.000	6.47	7.66	0.93	Indication signal (HIV; 0.18)
Pegaspargase	30	5.94	0.000	5.15	i.o.	0.64	Residual within-indication signal (ALL; 2.00)
Indinavir	22	60.95	0.000	54.45	62.16	0.79	Indication signal (HIV; 1.34)
Darunavir	21	3.19	0.000	3.40	3.24	0.72	Indication signal (HIV; 0.08)
Stavudine	19	19.92	0.000	21.31	22.30	1.19	Indication signal (HIV; 0.43)
Tenofovir (salt unspecified)‡	19	3.25	0.174	3.61	3.83	0.66	Indication signal (HIV; 0.09)
Adefovir	16	8.00	0.000	8.56	8.81	n.i.	Indication signal (HBV; 1.35)
Interferon alfa-2b	14	3.84	0.000	4.81	4.33	0.72	Not evaluable within viral hepatitis
Arsenic trioxide	14	9.35	0.417	8.79	11.35	n.i.	Residual within-indication signal (APL; 2.72)
Satralizumab	11	6.53	0.000	8.73	i.o.	0.57	Indication signal (NMOSD; 1.88)
Eliglustat	10	5.68	0.000	6.85	i.o.	n.i.	Indication signal (Gaucher; 0.27)

a, number of adjudicated scheme cases with the drug as suspect; ROR, reporting odds ratio (all reporters); JPI, jaw-propensity index; HCP ROR, health-professional-only ROR; GC− ROR, stratified ROR, in the glucocorticoid-unexposed stratum; α, Weibull shape parameter of time-to-onset. n.e., not evaluable (GC− stratum < 3 cases, as the drug is the glucocorticoid exposure); i.o., interaction-only stratum (3 ≤ a (GC−) < 10; pre-specified rule, no point estimate reported); n.i., insufficient onset data, fewer than five attributable onset records once matched reports are counted once, to fit a Weibull distribution. Methylprednisolone, triamcinolone, and dexamethasone are glucocorticoids; their GC− entries arise from reports in which the drug was not flagged as systemic-GC-exposed (local route formulations or incomplete flagging) and do not indicate a GC-independent association. Final adjudication follows the indication-restricted analysis and is one of glucocorticoid core, residual within-indication signal (within-indication ROR, with lower 95% bound above 1), indication signal (lower bound at or below 1, no residual excess detected), not evaluable within indication, or co-prescription marker. †Cholecalciferol is retained for transparency but classified as an antiresorptive co-prescription marker (fixed-dose alendronate + vitamin D3 combinations; JPI, 0.47, immediately below the 0.50 threshold), not a candidate cause. ‡Salt-unspecified tenofovir reports; the tenofovir disoproxil signal itself is litigation-inflated and excluded by the reporter restriction, and this small, health-professional-stable residual is adjudicated within the HIV indication.

## 3 Results

### 3.1 Descriptive analysis and case definition validity

The adjudicated scheme (Scheme C) yielded 24,087 non-jaw osteonecrosis cases (conservative Scheme A 1,521; permissive Scheme B 33,964). The adjudicated cases carried the demographic signature of genuine non-jaw osteonecrosis. They were younger (median age 53 versus 70 years), more often male (47.7% versus 10.2% among reports with a known sex), and more glucocorticoid-enriched (19.5% versus 13.7%) than an osteoporosis comparator, the opposite of the jaw-ONJ profile, confirming internal validity (Table 1; Figure 1; Supplementary Table S7).

### 3.2 Full-spectrum scan and the jaw-propensity index

The scan returned 47 four-criterion signals in the adjudicated scheme (Supplementary Table S8). The jaw-propensity index separated them cleanly. Denosumab (JPI 0.94), risedronate (0.78), zoledronic acid (0.71), ibandronate (0.65), pamidronate (0.55), and alendronate (0.502) clustered at the jaw-contaminated end, whereas tenofovir disoproxil (0.001), the glucocorticoids (0.00–0.28), and the transplant/Gaucher drugs (< 0.12) seldom carried the jaw term (Figures 2,3). The 0.50 threshold classified all six jaw-centered reference drugs against all six systemic glucocorticoids in the signal set in agreement with established knowledge (Youden J = 1.0), a consistency check rather than a validation (Section 2, Materials and Methods). The two-directional sweep showed where the threshold matters and where it does not (Supplementary Table S2; Supplementary Figure S1). The eight drugs in the final core (six glucocorticoids together with asparaginase and pegaspargase; maximum JPI 0.28) never changed class at any threshold between 0.30 and 0.70. Lowering the threshold to 0.30–0.40 removed arsenic trioxide and cholecalciferol (25 survivors), one of which, arsenic trioxide, is among the unresolved residuals, so that count is threshold-dependent even though the core is not, and 0.45 removed cholecalciferol alone (26). Increasing it to 0.55 re-admitted alendronate (28), 0.60 added pamidronate (29), and 0.65–0.70 added ibandronate (30). The threshold moves the borderline antiresorptives whose FAERS JPI lies between 0.50 and 0.65 in and out of the set, and a lower threshold also removes two drugs outside the core. It never changes the class of a core drug, which is exactly the class the index exists to detect, and their jaw-centeredness rests on evidence independent of the FAERS index because alendronate’s JPI is 0.66 in Canada Vigilance and 0.98 in JADER, pamidronate’s 0.67 and 0.97, and zoledronic acid’s 0.74 and 0.99 (Supplementary Table S2b), the onset profiles of pamidronate and zoledronic acid are delayed, and their reports arise from oncology populations. A conservative-stratum sensitivity analysis addressed the concern that rule R3 might delete genuine antiresorptive non-jaw cases (Supplementary Table S9). By construction, R1–R3 operate only on site-unspecified reports, so a report carrying an explicit non-jaw term can never be excluded by them; and in the R3-free Scheme A stratum, the antiresorptives indeed remain disproportional (pamidronate ROR 602.3, zoledronic acid 128.5, alendronate 12.3). Those explicit-term reports, however, are carried almost entirely by the site-silent ASEPTIC NECROSIS BONE term (pamidronate 141 of 146; zoledronic acid 401 of 415), co-report an explicit jaw term in fewer than 4% (3.4% and 0.7%). They arise from multiple myeloma, bone-metastasis, and metastatic breast cancer populations (71% and 76% with an oncology indication), precisely the populations in which jaw osteonecrosis occurs, indicating that the nominally explicit aseptic-necrosis vocabulary is itself site-ambiguous in practice and largely recodes jaw events from the litigation-era oncology population rather than marking a verified extra-oral phenotype. After jaw exclusion (41 of 47) and reporter restriction (33 of 47), 27 signals passed both filters; this intermediate set is listed in Table 2 together with the final adjudication of each drug. One member is transparently a co-prescription marker rather than a candidate cause. Cholecalciferol (vitamin D3, ROR 23.0, JPI 0.47) rides on its fixed-dose combinations with alendronate and on routine co-prescription with antiresorptives, sits immediately below the jaw threshold, and was excluded *a priori* from the negative-control set for exactly this reason (Section 2, Materials and Methods); it is retained in Table 2 for transparency and flagged accordingly.

**FIGURE 2:**
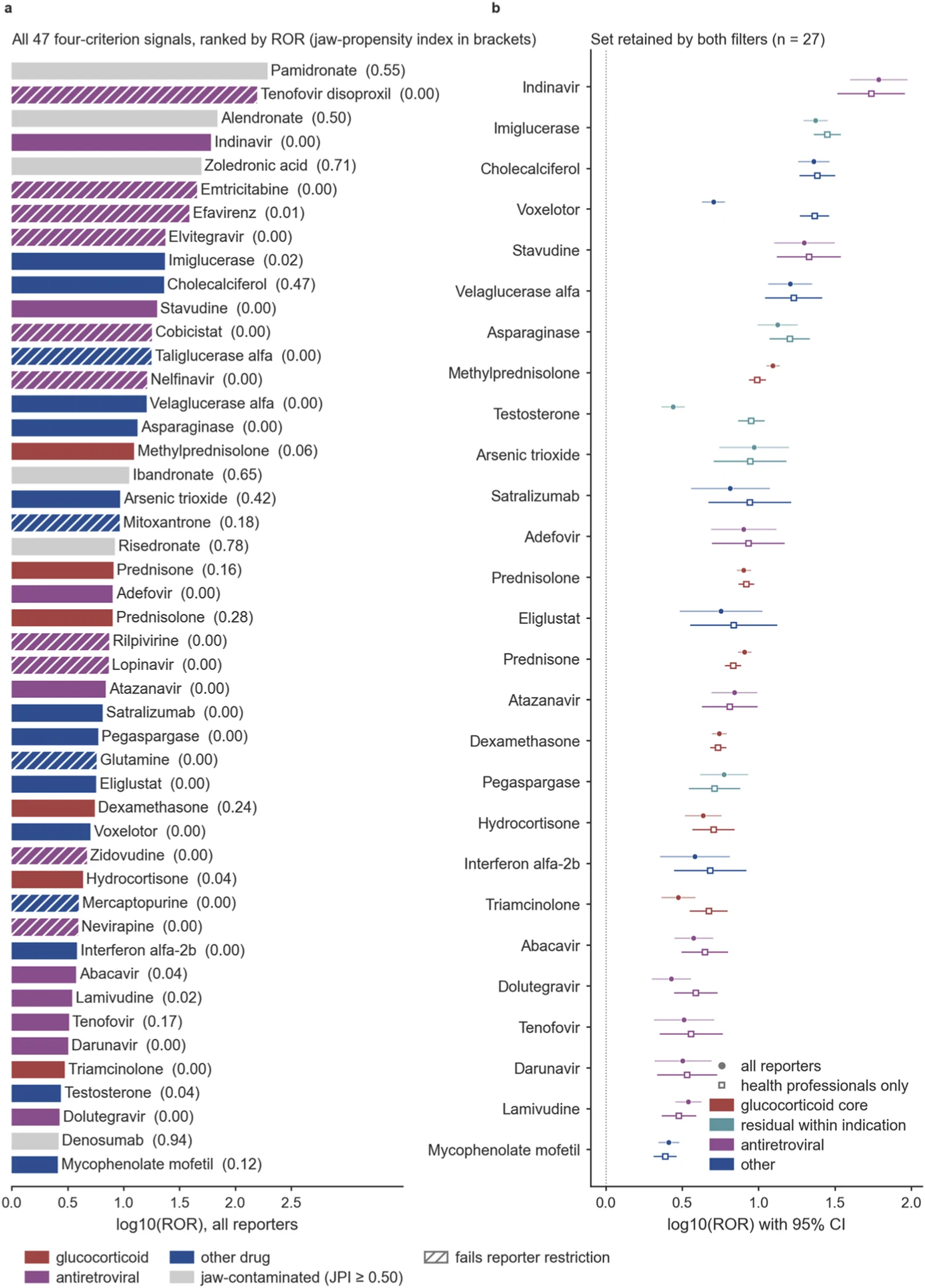
Full-spectrum disproportionality scan. **(a)** All 47 four-criterion signals of the adjudicated scheme, ranked by log_10_ reporting odds ratio (ROR, all reporters), each labeled with its jaw-propensity index (JPI, the share of the drug’s osteonecrosis-family reports coded as jaw). Color marks the systemic glucocorticoids (red), the antiretrovirals (violet), other non-jaw signals with JPI < 0.50 (blue), and jaw-contaminated signals with JPI ≥ 0.50 (gray); hatching marks signals that fail the reporter restriction (health-professional-only ROR not significant under all four criteria or below half the all-reporter ROR). **(b)** The 27-drug intermediate set that passes both filters, showing the all-reporter ROR (filled circles) and the health-professional-only ROR (open squares) with 95% confidence intervals; teal marks the residual within-indication signals (testosterone, imiglucerase, asparaginase, pegaspargase, and arsenic trioxide), and violet marks the antiretrovirals, a pharmacological class label. The antiretrovirals shown in this panel have passed the reporter restriction, so the color does not by itself mark litigation-recruited reporting. The dotted line marks ROR = 1. CI, confidence interval.

**FIGURE 3:**
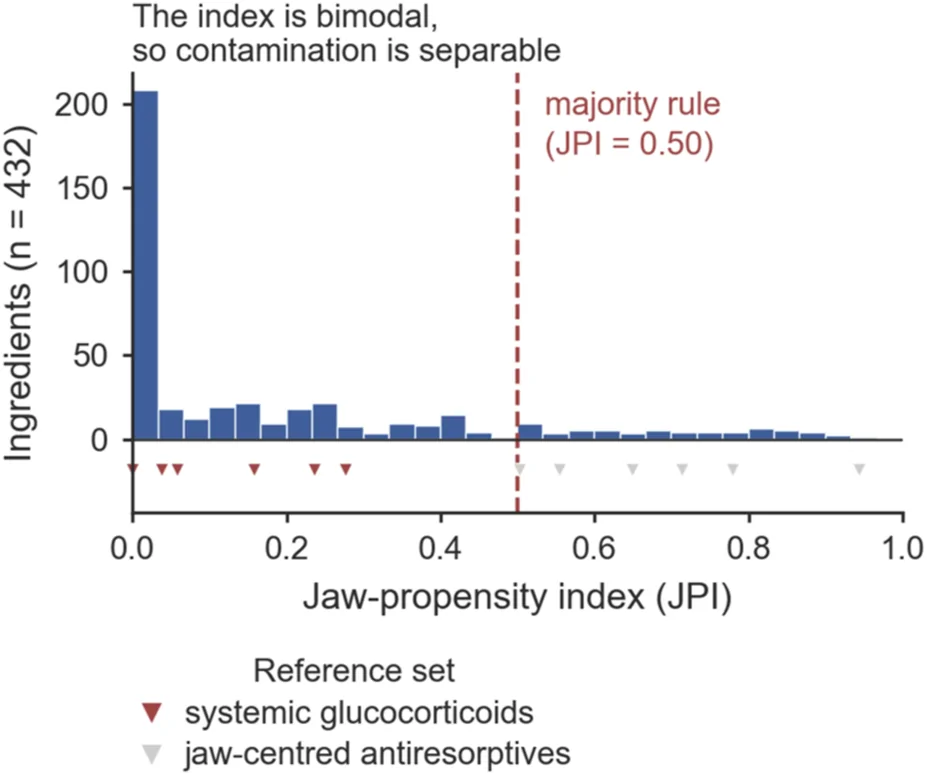
Jaw-propensity index (JPI) distribution. Histogram of the JPI (share of a drug’s osteonecrosis-family reports carrying the explicit OSTEONECROSIS OF JAW term) across all ingredients with at least three adjudicated scheme cases, with the pre-specified 0.50 majority rule threshold (dashed line) separating jaw-dominated from extra-oral reporting. ONJ, osteonecrosis of the jaw.

### 3.3 Reporting bias and the antiviral headline

The most extreme sensitivity involved tenofovir disoproxil, with an ROR of 157.9 (95% CI 153.4–162.6) in all reports but 6.33 (5.38–7.45) in health-professional-only reports (ratio 0.040), with only 148 of 7,027 cases reported by professionals and 5,937 (84.5%) filed under the lawyer code. Emtricitabine (45.8 → 4.79, 4.07–5.64; 79.1% lawyer-sourced) and efavirenz (38.9 → 2.22, 1.36–3.63, losing significance; 80.4% lawyer-sourced) behaved identically. A signal carried almost entirely by non-professional reports is the signature of litigation-driven solicitation (Figure 4; Table 3; Supplementary Table S8). Two further observations characterize the litigation channel. First, lawyer-sourced reports accounted for 25.8% of all adjudicated cases against 2.6% of the deduplicated corpus, a ten-fold enrichment of the endpoint. Second, excluding reports filed under the lawyer code left 46 of the 47 signals as four-criterion (rilpivirine alone dropped out; median ROR ratio 1.31), whereas restriction to health professional reporters left only 35 of them as four-criterion signals and exclusion of consumers left only 32. Because lawyer-solicited reports reach FAERS predominantly through the consumer channel, the lawyer code alone cannot identify them, which is why the health professional restriction and the cross-jurisdiction comparison were pre-specified as the litigation screen. The reporter filter also removed glutamine (22 cases, ROR 5.73, 3.77–8.72), a drug licensed for sickle-cell disease whose cases were all consumer-reported with no health professional report, whereas voxelotor, licensed for the same disease, was reported more strongly by professionals than by all reporters (HCP ROR 23.2, 18.6–29.0, versus 5.06; 0.8% lawyer-sourced), and testosterone, despite its history of unrelated mass tort litigation, was lawyer-sourced in only 2.4% of cases and retained a stable professional signal (8.94, 7.31–10.9).

**FIGURE 4:**
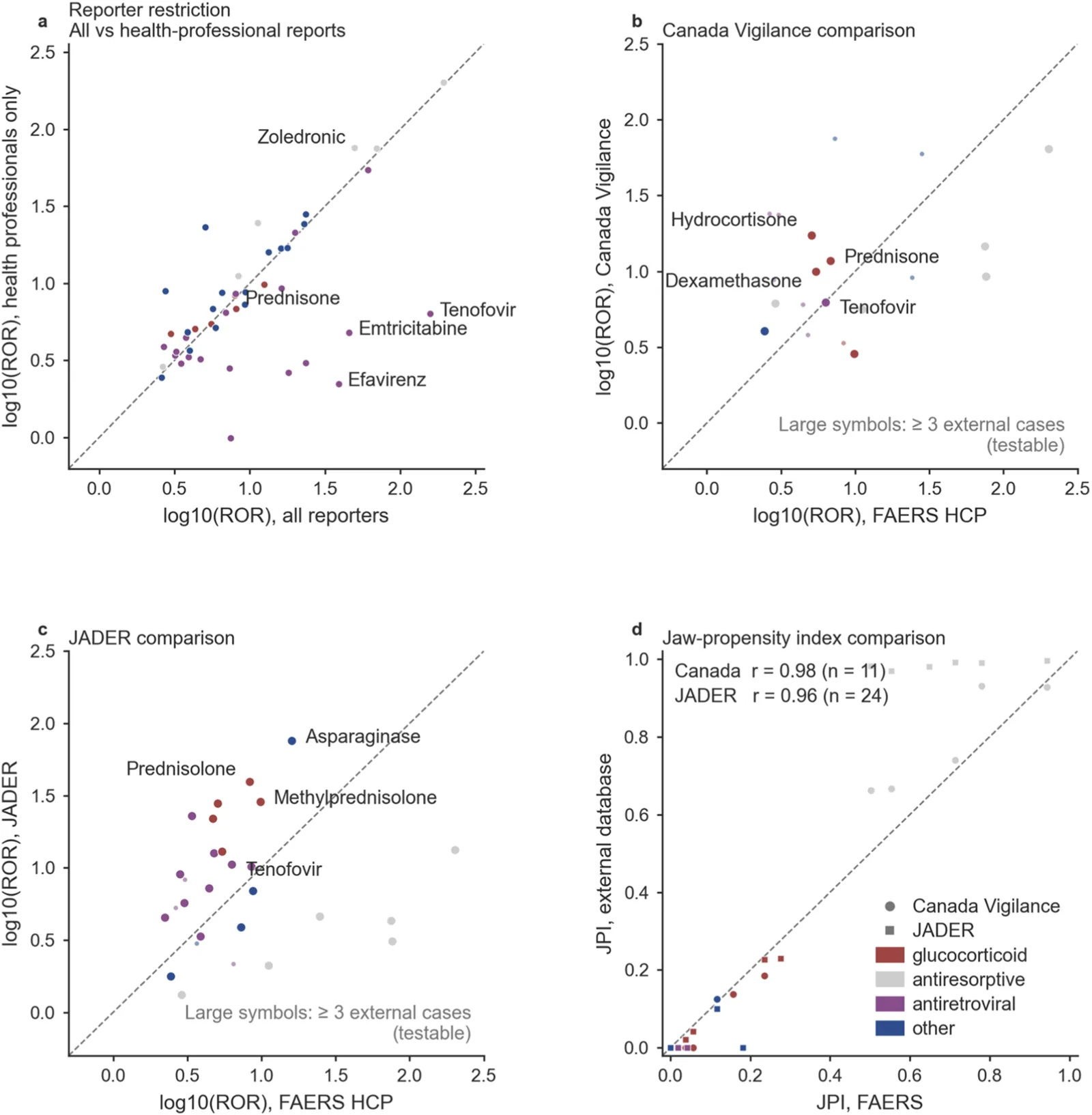
Reporting bias and external validation. **(a)** Reporting odds ratio (ROR) of the 47 four-criterion signals under reporter restriction, with all reporters on the x-axis and only health professional reporters (HCPs) on the y-axis, both log_10_; red, glucocorticoids; violet, antiretrovirals; gray, antiresorptives; blue, other drugs; the dashed line is the identity. Signals far below the identity (tenofovir disoproxil, emtricitabine, and efavirenz) are carried by non-professional reporting. **(b,c)** Cross-database replication, showing the FAERS health-professional-only ROR against the ROR recomputed in Canada Vigilance **(b)** and JADER **(c)**; large symbols mark drugs with ≥ 3 external cases (testable); small symbols mark drugs with 1–2 external cases (not testable, shown for completeness). **(d)** Replication of the jaw-propensity index (JPI), showing the FAERS JPI against the JPI recomputed in Canada Vigilance (circles) and JADER (squares) for drugs with ≥ 3 external cases (Pearson r = 0.985, n = 11; r = 0.960, n = 24). FAERS, U.S. Food and Drug Administration Adverse Event Reporting System; JADER, Japanese Adverse Drug Event Report database.

**TABLE 3:** Reporting-bias sensitivity and external validation.

Drug	FAERS ROR, all (95% CI)	FAERS ROR, HCP (95% CI)	Ratio	Canada ROR (95% CI) (a)	JADER ROR (95% CI) (a)	Replicated (Canada/JADER)
Tenofovir disoproxil	157.9 (153.4–162.6)	6.33 (5.38–7.45)	0.040	6.24 (2.00–19.4) (3)	10.5 (6.60–16.8) (18)	Directionally consistent*/directionally consistent*
Emtricitabine	45.8 (43.9–47.7)	4.79 (4.07–5.64)	0.105	3.82 (0.54–27.2) (1)	12.7 (8.22–19.6) (21)	n.t./yes†
Efavirenz	38.9 (35.8–42.3)	2.22 (1.36–3.63)	0.057	n.a.	4.53 (1.46–14.1) (3)	n.t./no
Prednisone	8.11 (7.33–8.96)	6.81 (6.04–7.68)	0.840	11.7 (9.24–14.9) (81)	n.t. (0)	Yes/n.t.‡
Dexamethasone	5.52 (4.93–6.17)	5.42 (4.80–6.13)	0.983	10.0 (6.52–15.4) (22)	13.0 (11.1–15.2) (184)	Yes/yes
Hydrocortisone	4.33 (3.29–5.70)	5.06 (3.68–6.97)	1.169	17.3 (10.3–29.0) (15)	28.0 (20.8–37.8) (46)	Yes/yes
Methylprednisolone	12.4 (11.2–13.8)	9.81 (8.64–11.1)	0.790	2.87 (1.07–7.69) (4)	28.7 (24.7–33.3) (207)	n.s/yes
Mycophenolate mofetil	2.58 (2.21–3.01)	2.44 (2.05–2.91)	0.947	4.05 (1.92–8.55) (7)	1.78 (1.12–2.84) (18)	n.s/n.s.

ROR, reporting odds ratio in the adjudicated scheme with 95% confidence interval; HCP, health-professional reporters only; (a), osteonecrosis case count in that database. For Replicated (Canada/JADER): yes, replicated under all four criteria; n.s, directionally consistent but not meeting all four criteria in that database; no, not replicated; n.t., not testable (< 3 cases); n.a., not applicable. For tenofovir disoproxil, the Canadian estimate rests on three cases, and the Japanese estimate rests on 18; both lie more than an order of magnitude below the FAERS all-reporter ROR and are directionally consistent with the FAERS health-professional-only estimate, which is the litigation attribution being tested, not a conventional replication. Within HIV-indication reports, the JADER estimate is 2.75 (1.26–6.00). †Emtricitabine meets all four criteria in JADER, but it is co-formulated with tenofovir disoproxil, so its external signal tracks the tenofovir association rather than an independent emtricitabine effect; efavirenz is likewise co-formulated. ‡Prednisone is largely replaced by prednisolone in Japan, hence zero JADER cases.

### 3.4 External validation in Canada Vigilance and JADER

Canada Vigilance provided a first comparison in a reporting system in which the recorded lawyer channel carries a far smaller share of this endpoint than the American one (2 lawyer-filed of 1,127 tenofovir reports, and none of the 496 Canadian osteonecrosis cases of any drug). The tenofovir disoproxil ROR was 6.24 (95% CI 2.00–19.4), an estimate resting on only three Canadian cases among 1,127 tenofovir reports. It is, therefore, imprecise and cannot be read as a replication, but it is directionally consistent with the FAERS health-professional-only estimate (6.33, 5.38–7.45) and is approximately 25-fold below the all-reporter estimate (157.9). JADER, the better-powered second database, returned 10.5 (6.60–16.8) from 18 cases among 1,332 tenofovir reports, again far below the FAERS all-reporter estimate but modestly above the health-professional-only estimate. Because JADER records the underlying disease, the indication restriction could be applied to the Japanese estimate, and within HIV-indication reports (n = 3,598; HIV itself ROR 5.62, 3.81–8.30, for 骨壊死), the tenofovir disoproxil ROR was 2.75 (1.26–6.00; 11 cases) and that of emtricitabine 3.97 (1.80–8.78). The same restriction applied to FAERS leaves tenofovir disoproxil at 27.5 (25.2–30.1) because the litigation-recruited reports lie inside the HIV stratum itself, and 94% of the osteonecrosis cases carrying an HIV indication name tenofovir disoproxil or one of its co-formulated partners. Holding the disease constant, therefore, does not dissolve the discrepancy between the two jurisdictions. It is compatible with an effect of the reporting context, although it cannot separate that from differences in disease stage, treatment era, coding, or reporting structure between the two countries. The Japanese within-HIV estimate is an order of magnitude smaller than the American one (Supplementary Table S10). The strength of the litigation attribution lies in the convergence of the Canadian and Japanese estimates, none of which approaches the FAERS all-reporter value, rather than in the precision of any single database; a residual tenofovir–osteonecrosis association of the modest size seen in those estimates is not excluded and is taken up in Section 4, Discussion. The concordance of the whole survivor set was then examined. Only 5 of the 27 intermediate set drugs had ≥ 3 Canadian cases and were thus externally testable. Three of those replicated under all four criteria, namely prednisone (ROR 11.7, 9.24–14.9), dexamethasone (10.0, 6.52–15.4) and hydrocortisone (17.3, 10.3–29.0), while methylprednisolone (2.87, 1.07–7.69; 4 cases) and mycophenolate (4.05, 1.92–8.55; 7 cases) were directionally consistent but did not meet all four criteria, the external case counts being too small to do so. JADER extended the test to 13 of the 27 drugs with ≥ 3 Japanese cases, of which nine replicated under all four criteria (methylprednisolone, prednisolone, dexamethasone, lamivudine, triamcinolone, hydrocortisone, abacavir, asparaginase, and darunavir). The glucocorticoids reproduced with large effect sizes in JADER, led by prednisolone (ROR 39.3, 35.3–43.8; 729 cases), methylprednisolone (28.7, 24.7–33.3; 207) and dexamethasone (13.0, 11.1–15.2; 184), and the glucocorticoid class pooled ROR was 54.4 (48.3–61.3) in JADER and 10.3 (8.28–12.8) in Canada, whereas the bisphosphonates and denosumab again showed high jaw propensity in both databases. Asparaginase, the strongest non-glucocorticoid residual, replicated in JADER with ROR 75.7 (61.4–93.3) from 103 cases. The jaw-propensity index replicated across databases with Pearson r = 0.985 (Canada) and r = 0.960 (JADER), each computed over the drugs with ≥ 3 osteonecrosis cases in the external database (n = 11 and n = 24) (Figure 4; Table 3; Supplementary Tables S6, S11). The three databases differ structurally, in period (Canada Vigilance to November 2024; FAERS to 2026Q2; JADER to the end of Japanese fiscal-year 2025, that is to March 2026, in the June 2026 release), in reporting channel (consumers filed 21% of Canadian reports and 16% of its osteonecrosis cases; a consumer qualification appears on 8.4% of JADER reports and a lawyer qualification appears on 0.02%), and in coding (MedDRA versus MedDRA/J). Supplementary Table S6 sets these characteristics next to the concordance observed.

### 3.5 Indication-restricted analysis

To test whether the surviving signals were carried by their home diseases, the ROR of every non-glucocorticoid drug of the intermediate set (21 drugs) was recomputed within reports carrying its home indication, together with tenofovir disoproxil, zidovudine, nevirapine, and taliglucerase alfa, which are analyzed for continuity with the submitted version although they are not in the intermediate set (25 drugs in all) (Table 4; Supplementary Table S12). Every home indication except chronic viral hepatitis (0.88, 0.76–1.03) was itself associated with osteonecrosis, strongly for HIV infection (ROR 68.5, 95% CI 66.6–70.4), Gaucher disease (19.8, 95% CI 17.0–23.0), hypogonadism (8.37), acute promyelocytic leukemia (7.36), osteoporosis (6.50), sickle-cell disease (6.08), and hepatitis B (5.97), and modestly for neuromyelitis optica (4.57), acute lymphoblastic leukemia (2.73), and transplantation (2.11), so each carries confounding potential and the modest ones set a correspondingly low bar for a drug to clear. Within-indication, mycophenolate fell to ROR 0.89 (95% CI 0.68–1.16), with no residual excess over the transplant baseline and is adjudicated an indication signal. Voxelotor likewise fell to the sickle-cell baseline with no residual excess (0.97, 0.71–1.32), accounting for 64% of the reports and 64% of the osteonecrosis cases of that stratum, so within the disease it is reported with osteonecrosis no more often than the other sickle-cell therapies. Among the Gaucher-directed therapies, which are the enzyme replacements imiglucerase, velaglucerase alfa, and taliglucerase alfa together with the substrate-reduction agent eliglustat, the estimates formed a gradient (imiglucerase 1.72 (1.26–2.35), velaglucerase alfa 0.85 (0.58–1.25), and eliglustat 0.27 (0.14–0.53)), a pattern compatible with the oldest and most widely used agent being given to the most severely affected patients, although neither treatment duration nor disease severity is recorded in these data, and neither was measured here. Asparaginase remained clearly elevated within the leukemia indication (ROR 7.14, 5.10–10.0; 38 of its 43 cases carrying the indication), as did its pegylated form pegaspargase (2.00, 1.26–3.17). Tenofovir disoproxil, by contrast, remained at 27.5 (25.2–30.1) inside the HIV stratum, because the litigation-recruited reports lie within that stratum rather than outside it (Tables 3 and 4). Of the eight antiretrovirals of the intermediate set, 63%–95% of cases carried the HIV indication, and all eight fell to or below the HIV baseline, several of them far below it, which is what a stratum dominated by the litigation cluster produces (within-HIV ROR 0.06–1.34, every lower 95% bound ≤ 1), and are adjudicated as indication signals. Because 94% of the HIV-indication osteonecrosis cases name tenofovir disoproxil or a co-formulated partner, the HIV stratum itself is dominated by the litigation cluster and the within-HIV ratios of the other antiretrovirals are biased toward the null. After removing every report naming a litigated antiretroviral from the stratum (75,386 reports, 477 cases), the within-HIV RORs of the remaining antiretrovirals were 0.49–12.2, and only atazanavir (1.62, 1.08–2.44), indinavir (12.2, 7.49–19.9), and stavudine (3.83, 2.15–6.84) retained a lower bound above 1 (Supplementary Table S12c). Among the remaining low-count drugs, adefovir within hepatitis B (1.35, 0.74–2.45) and satralizumab within neuromyelitis optica spectrum disorder (1.88, 0.92–3.86) showed no residual excess over the home-disease baseline. Testosterone within hypogonadism/androgen deficiency (4.60, 1.96–10.8) and arsenic trioxide within acute promyelocytic leukemia (2.72, 1.24–5.99) remained elevated within the home indication. Interferon alfa-2b within chronic viral hepatitis (B or C) (no within-indication case) was not evaluable. Cholecalciferol within osteoporosis/vitamin D deficiency gave ROR 7.93 (5.96–10.6), consistent with its co-prescription with antiresorptives within that indication rather than with an independent effect (it also loses four-criterion status when antiresorptive reports are removed, Supplementary Table S5).

**TABLE 4:** Indication-restricted analysis.

Drug	Home indication	Indication ROR (95% CI)	n (ind)	a (ind)	Within-indication ROR (95% CI)	Verdict
Mycophenolate mofetil	Transplant/immunosuppression	2.11 (1.93–2.31)	28,676	63	0.89 (0.68–1.16)	Indication signal
Asparaginase	Acute lymphoblastic leukemia	2.73 (2.47–3.01)	1,879	38	7.14 (5.10–10.0)	Residual within-indication signal
Pegaspargase	Acute lymphoblastic leukemia	2.73 (2.47–3.01)	3,119	19	2.00 (1.26–3.17)	Residual within-indication signal
Tenofovir disoproxil (not in intermediate set)	HIV infection	68.5 (66.6–70.4)	49,416	6,831	27.5 (25.2–30.1)	Litigation-inflated (Table 3)
Tenofovir (salt unspecified)	HIV infection	68.5 (66.6–70.4)	3,515	18	0.09 (0.06–0.14)	Indication signal
Lamivudine	HIV infection	68.5 (66.6–70.4)	16,107	72	0.07 (0.06–0.09)	Indication signal
Abacavir	HIV infection	68.5 (66.6–70.4)	7,297	41	0.10 (0.07–0.13)	Indication signal
Dolutegravir	HIV infection	68.5 (66.6–70.4)	9,087	31	0.06 (0.04–0.08)	Indication signal
Zidovudine (not in intermediate set)	HIV infection	68.5 (66.6–70.4)	4,629	35	0.13 (0.09–0.18)	Indication signal
Atazanavir	HIV infection	68.5 (66.6–70.4)	2,491	25	0.18 (0.12–0.27)	Indication signal
Nevirapine (not in intermediate set)	HIV infection	68.5 (66.6–70.4)	4,989	27	0.09 (0.06–0.14)	Indication signal
Indinavir	HIV infection	68.5 (66.6–70.4)	258	18	1.34 (0.83–2.17)	Indication signal
Darunavir	HIV infection	68.5 (66.6–70.4)	3,308	15	0.08 (0.05–0.13)	Indication signal
Stavudine	HIV infection	68.5 (66.6–70.4)	513	12	0.43 (0.24–0.76)	Indication signal
Imiglucerase	Gaucher disease	19.8 (17.0–23.0)	3,901	110	1.72 (1.26–2.35)	Residual within-indication signal
Velaglucerase alfa	Gaucher disease	19.8 (17.0–23.0)	1,714	34	0.85 (0.58–1.25)	Indication signal
Taliglucerase alfa (not in intermediate set)	Gaucher disease	19.8 (17.0–23.0)	371	8	0.96 (0.47–1.96)	Indication signal
Eliglustat	Gaucher disease	19.8 (17.0–23.0)	1,275	9	0.27 (0.14–0.53)	Indication signal
Voxelotor	Sickle-cell disease	6.08 (5.23–7.06)	15,910	110	0.97 (0.71–1.32)	Indication signal
Testosterone	Hypogonadism/androgen deficiency	8.37 (6.33–11.1)	3,200	44	4.60 (1.96–10.8)	Residual within-indication signal
Adefovir	Hepatitis B	5.97 (4.98–7.14)	1,333	12	1.35 (0.74–2.45)	Indication signal
Interferon alfa-2b	Chronic viral hepatitis (B or C)	0.88 (0.76–1.03)	360	0	n.e.	n.e.
Satralizumab	Neuromyelitis optica spectrum disorder	4.57 (3.28–6.37)	1,298	11	1.88 (0.92–3.86)	Indication signal
Arsenic trioxide	Acute promyelocytic leukemia	7.36 (4.97–10.9)	751	12	2.72 (1.24–5.99)	Residual within-indication signal
Cholecalciferol	Osteoporosis/vitamin D deficiency	6.50 (6.21–6.81)	984	51	7.93 (5.96–10.6)	Co-prescription marker

Indication ROR, the ROR of the indication itself for osteonecrosis over the whole universe (its confounding potential), with a 95% confidence interval. n (ind), reports with the drug as suspect that carry the indication; a (ind), osteonecrosis cases among them. Within-indication ROR is the ROR of the drug recomputed inside reports carrying the indication, holding the disease baseline constant. Verdict follows the pre-specified rule, being a residual within-indication signal when the lower 95% bound exceeds 1, an indication signal when it does not, and n.e. (not evaluable) when there are fewer than three within-indication cases or a zero cell. Two rows carry a verdict from elsewhere in the analysis. Tenofovir disoproxil is not a member of the intermediate set, having been excluded by reporter restriction, and its within-indication elevation reflects the litigation reports that lie inside the HIV stratum (Table 3); cholecalciferol is an antiresorptive co-prescription marker (Table 2). The residuals differ in evidential weight, and only the asparaginase class also has a specific mechanism and an independent external replication.

In total, of the 20 non-glucocorticoid drugs adjudicated within their indication (cholecalciferol being classified *a priori* as a co-prescription marker), 14 were indication signals, five residual within-indication signals, and one not evaluable. The residuals are not equivalent in evidential weight because only the asparaginase class combines a large within-indication estimate with a specific mechanism and an independent external replication, whereas testosterone, arsenic trioxide, and imiglucerase are modest estimates in strata where channeling to more severely affected patients or glucocorticoid co-treatment is the more economical explanation (Section 4, Discussion).

### 3.6 Empirical null calibration

All 34 negative controls (Supplementary Table S4) showed ROR < 1 (median 0.16), confirming severe competitive masking, whereby the bisphosphonate, tenofovir, and glucocorticoid signals inflate the background event rate and depress the ROR of every other drug. The fitted systematic-error null (μ = −1.70, σ = 0.62 on the natural-log ROR scale) locates the negative-control distribution ≈ 5.5-fold below the unbiased expectation (exp (−μ)), a direct measure of the masking depth. Re-testing every signal against this empirical null rather than the theoretical null left all 47 significant, which follows from the null being centered below 1 and is therefore a consistency check rather than an independent hurdle. The unmasking analysis made the mechanism explicit (Supplementary Table S5; Supplementary Figure S3). Removing the 383,790 reports in which an antiresorptive or a litigated antiretroviral was a suspect drug (13,986 cases, 58% of all cases) raised the median ROR of the same 34 controls from 0.16 to 0.38 (94% still below 1), and removing the 188,025 further reports that name a systemic glucocorticoid as a suspect drug, 571,815 in all, raised it to 0.44 (88% of the 34 controls still below 1), so the three clusters account for roughly half of the masking depth on the log scale and the remaining signals for the rest. As expected for drugs outside the removed clusters, unmasking rescales the ROR of every remaining drug by an almost constant factor (intermediate set median 2.35, range 2.23–2.96; prednisone 8.11 → 19.4, asparaginase 13.3 → 31.3, voxelotor 5.06 → 11.9), so the ranking of the intermediate set was unchanged; what changes is which drugs cross the four-criterion thresholds. Two members of the intermediate set lost four-criterion status at stage 1, cholecalciferol and tenofovir, because their cases were carried by reports that also named an antiresorptive or tenofovir disoproxil, which supports their interpretation as co-prescription and co-formulation markers. Conversely, 43 recorded drug names outside the primary signal set newly met all four criteria at stage 1, and 45 met all four criteria at stage 2. These are names as recorded, not deduplicated ingredients because a few are brand entries of a drug already listed under its ingredient name. Of these drugs, 84% are drugs used in populations in which glucocorticoid exposure, malignancy, or hemoglobinopathy is the plausible driver, namely transplant/autoimmune immunosuppressant, six drugs (azathioprine, belumosudil, cyclosporine, lymphocyte immune globulin (anti-thymocyte globulin), sirolimus and tacrolimus); leukemia/lymphoma/myeloma chemotherapy, 13 drugs (blinatumomab, cyclophosphamide, cytarabine, doxorubicin, etoposide, fludarabine, mabthera (rituximab, brand entry), melphalan, methotrexate, rituximab, thalidomide, tretinoin and vincristine); breast/prostate cancer endocrine or targeted therapy, eight drugs (anastrozole, bevacizumab, exemestane, fulvestrant, goserelin, imatinib, letrozole and taxotere (docetaxel, brand entry)); sickle-cell disease/thalassaemia, three drugs (crizanlizumab, deferasirox and hydroxyurea); lysosomal storage disease therapy, one drug (miglustat); osteoporosis anabolic (antiresorptive co-prescription), one drug (romosozumab); rheumatology/neurology immunomodulator, four drugs (hydroxychloroquine, interferon beta-1a, leflunomide and upadacitinib); antiretroviral booster (HIV), one drug (ritonavir); growth hormone, one drug (somatropin); other (opioid analgesic), one drug (duragesic (fentanyl, brand entry)); other (unmapped product name), one drug (isopropyl alcohol); other, three drugs (betamethasone, raloxifene, and raltegravir). These unmasked associations were not carried through the full adjudication pipeline, which was designed around the primary signal set; they are listed as hypothesis-generating in Supplementary Table S5, and their composition is itself evidence that the residual landscape of this endpoint is disease-driven. Both analyses show that the observed RORs depend on the composition of the reporting background rather than on the drug alone because the negative-control distribution sits well below ROR = 1. Removing the dominant clusters moves every remaining estimate in the same direction. That is a statement about the comparator, not a bound on any causal quantity, and we do not apply the shift as a correction to individual drugs (Figure 5).

**FIGURE 5:**
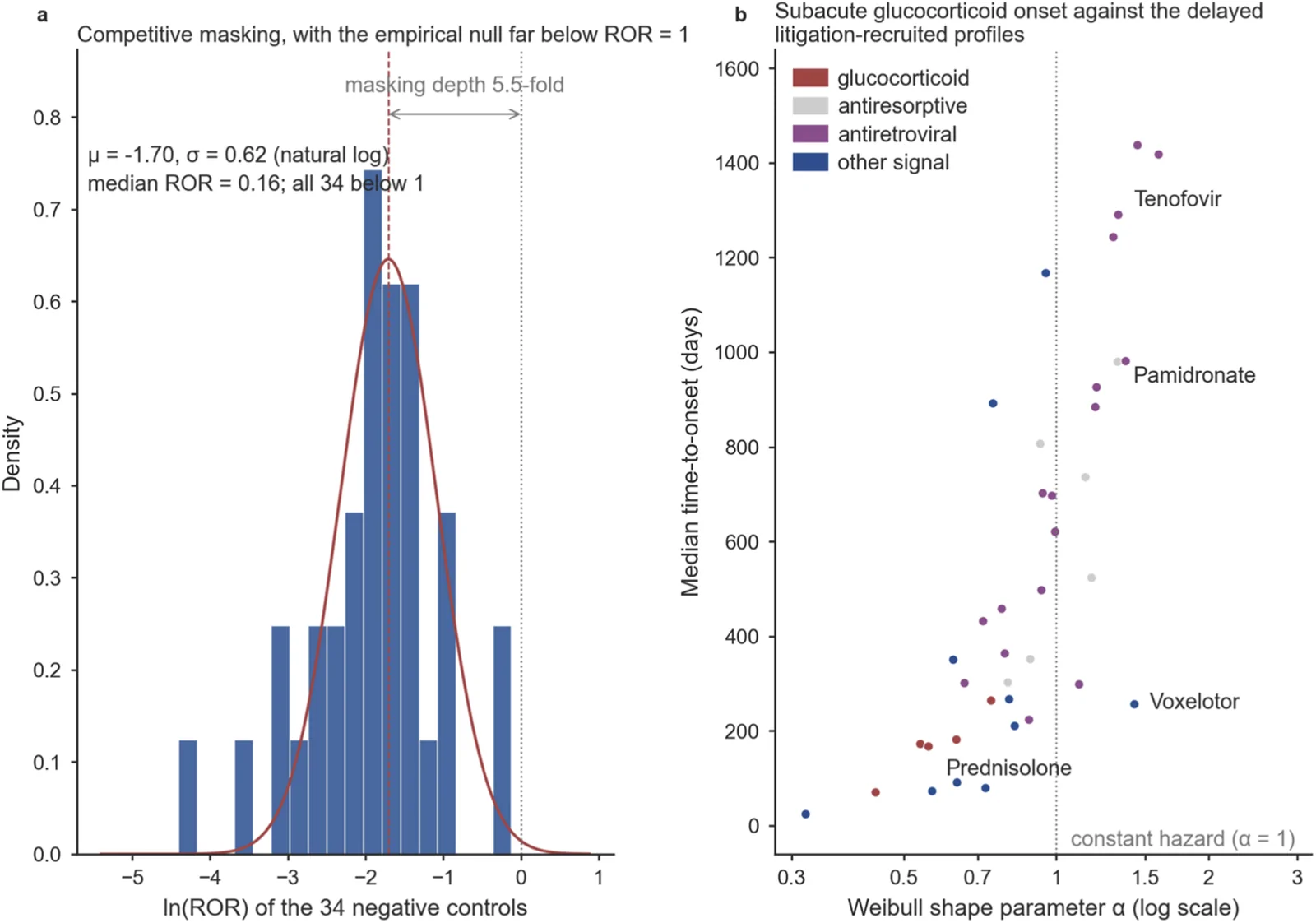
Empirical null and time-to-onset. **(a)** Distribution of the natural-log reporting odds ratio (ln ROR) of the 34 negative controls with the fitted systematic-error null N (μ, σ) (red curve; dashed line at μ), showing that the whole negative-control distribution sits below ROR = 1 (dotted line), the signature of competitive masking; exp (−μ), the masking depth, and the median ROR are given in the panel. **(b)** Weibull shape parameter α (log scale) of the time-to-onset distribution versus median time-to-onset for every drug with at least five attributable onset records, separating the subacute glucocorticoid signature (α < 1, red) from the delayed antiretroviral signature (violet), with the antiresorptives (gray) falling on both sides of α = 1; blue, other signals; the dotted line marks α = 1 (constant hazard). Every drug with at least five attributable onset records is plotted; the complete per-drug results, including the drugs whose shape parameter rests on few distinct onset values, are given in Supplementary Table S13.

### 3.7 Glucocorticoid stratification (H2)

The glucocorticoid stratification showed that the glucocorticoids themselves (prednisone, methylprednisolone, dexamethasone, prednisolone, hydrocortisone, triamcinolone) are trivially GC-dependent, being themselves the exposure. Contrary to H2, however, GC stratification did not collapse the non-glucocorticoid survivors, which remained GC-independent, the expected signature of disease confounding rather than glucocorticoid confounding. Seventeen of the 21 non-glucocorticoid survivors met the pre-specified threshold for a reportable GC-unexposed ROR (a (GC−) ≥ 10), and all of them had a GC-unexposed lower bound above 1; the four below the reporting threshold (asparaginase, pegaspargase, satralizumab, and eliglustat) fall under the interaction-only rule and are reported as directional only (Table 2). The strongest GC-independent signals were the disease markers indinavir (GC− ROR 62.2), imiglucerase (28.4), stavudine (22.3), and velaglucerase alfa (19.0), whereas the weakest was mycophenolate (GC− ROR 1.52, 95% CI 1.13–2.05; Firth-adjusted OR 1.48, 1.27–1.74), consistent with its role as a marker of the transplant population. The adversarial tipping-point analysis graded these calls rather than certifying them uniformly (Supplementary Table S3; Supplementary Figure S2). Of the 36 evaluable drugs, 29 were robust (tipping fraction ≥ 0.60, 2 of them beyond the 0.99 grid), 6 moderate, and 1 fragile. The disease markers would require most of their GC-negative cases to be reassigned before losing significance (imiglucerase 94%, indinavir 93%, velaglucerase alfa 87%, voxelotor 76%). In contrast, mycophenolate, the weakest association, would be explained away by misclassifying only 10% of its 43 GC-negative cases, or approximately five reports. Because systemic-GC co-exposure was recorded in 44.8% of ≥ 5-drug reports but only 5.3% of single-drug reports, under-recording of that size is plausible, so the mycophenolate call is fragile on this ground alone, independently anticipating the indication-restricted analysis and the final adjudication (Figure 6; Tables 4, 5).

**FIGURE 6:**
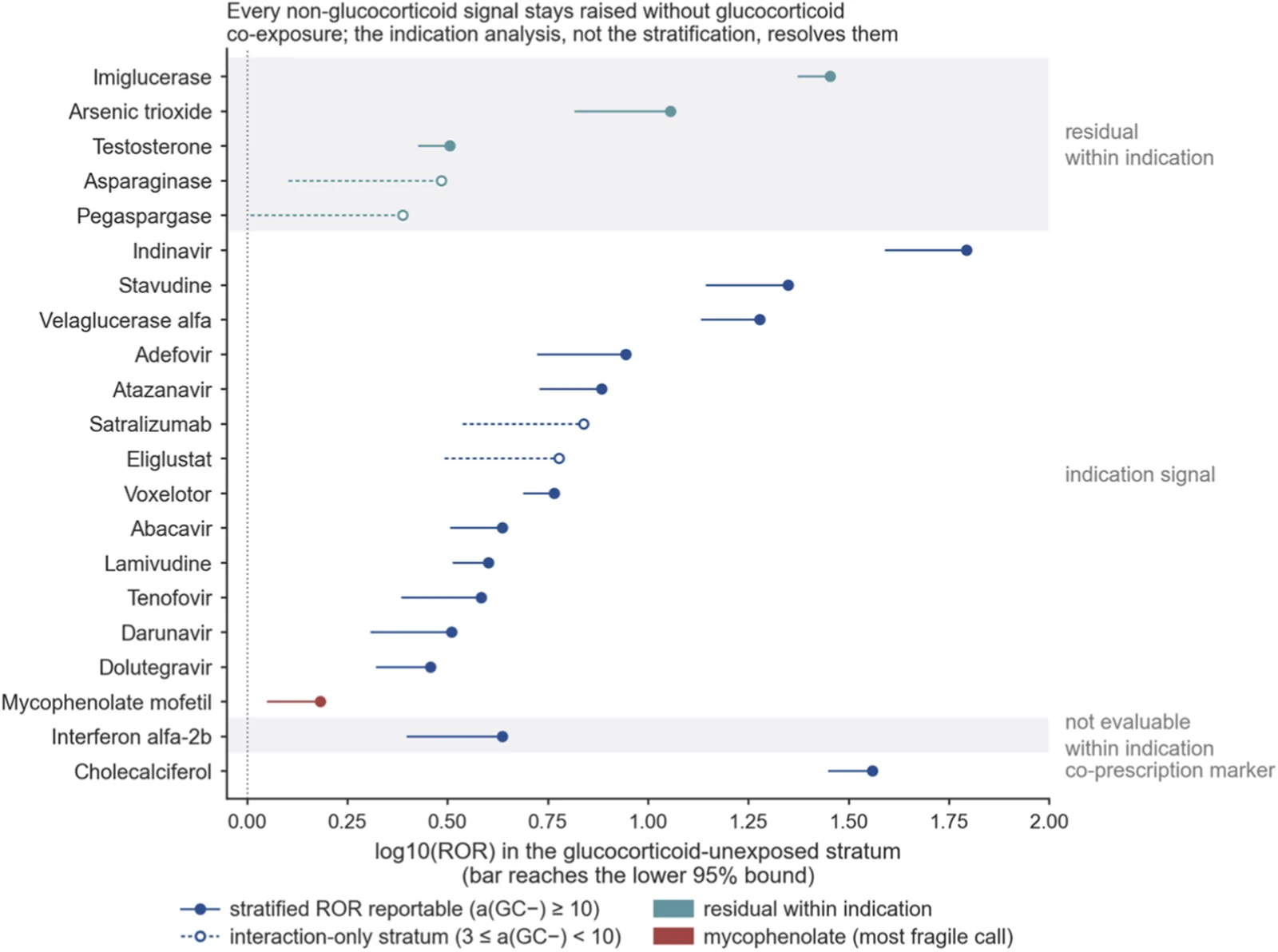
Glucocorticoid co-exposure stratification. Reporting odds ratio (ROR, log_10_) in the glucocorticoid-unexposed stratum for the 21 non-glucocorticoid drugs of the intermediate set, with the bar extending to the lower 95% confidence bound, and the dotted line at ROR = 1. Filled symbols mark drugs with a reportable stratified ROR (a (GC−) ≥ 10, pre-specified rule) and open symbols with dashed bars mark interaction-only strata (3 ≤ a (GC−) < 10), shown for direction only. Mycophenolate, the weakest association (tipping fraction 10%), is highlighted in red. GC, glucocorticoid.

**TABLE 5:** Glucocorticoid stratification and temporal signature.

Class	GC− ROR	Firth OR	Weibull α	Median onset	Interpretation
Glucocorticoids	n.e. (drug = GC)	n.a.	0.44–0.74	71–265 d	Subacute onset; the only class that survives every correction
Bisphosphonates	(Jaw-contaminated)	n.a.	0.89–1.32	353–980 d	Jaw ONJ, delayed for the intravenous agents
Antiretrovirals (litigated)	Elevated (litigation)	Tenofovir 226.19 (218.89–233.73)	1.30–1.45	1,244–1,438 d	Litigation-recruited
Mycophenolate	1.52 (1.13–2.05)	1.48 (1.27–1.74)	0.83	211 d	Transplant indication signal (excluded from final set)
Asparaginase	i.o.	n.a.	n.a.	n.a. d	ALL regimen with dexamethasone; residual within indication

n.e., not evaluable; n.a., not applicable. GC− ROR, reporting odds ratio in the glucocorticoid-unexposed stratum with its 95% CI, or i.o. where the stratum holds fewer than ten cases, and only the direction is reported; Firth OR, Firth-penalized odds ratio adjusted for age, sex, report year, and glucocorticoid co-exposure; ALL, acute lymphoblastic leukemia. Weibull α and median onset are the range across the drugs of the class, or the drug’s own value where the row names one drug (Supplementary Table S13).

### 3.8 Time-to-onset (H3)

The Weibull shape parameter separated the glucocorticoid profile from the litigation-recruited one at the level of their central estimates, although the per-drug intervals overlap and no formal between-class test was performed (Supplementary Table S13). Glucocorticoids showed subacute onset (α 0.44–0.74, fitted Weibull median 71–265 days), whereas the antiresorptives divided. Two of the six lay unambiguously in the increasing-hazard range, namely pamidronate α 1.32 (1.17–1.51) and zoledronic acid α 1.17 (1.11–1.24), matching the known natural history of jaw ONJ, while the remainder were not separable from constant hazard, namely risedronate α 1.14 (0.77–1.76), alendronate α 0.93 (0.84–1.02), ibandronate α 0.89 (0.75–1.07), and denosumab α 0.80 (0.65–1.02), so the delayed temporal signature corroborates the jaw contamination reading for the intravenous agents given in oncology rather than for the antiresorptive class as a whole. The jaw-centeredness of the oral agents rests on the propensity index and the reporting population rather than on timing. The litigation-heavy antiretrovirals showed the most delayed profile, namely tenofovir disoproxil (α 1.33, median 1,292 days), emtricitabine (1.30, 1,244 days) and efavirenz (1.45, 1,438 days), consistent with litigation-recruited late reports rather than pharmacology. In contrast, the surviving non-litigated antiretrovirals were temporally distinct from them (lamivudine α 0.99, dolutegravir 0.88, darunavir 0.72). Asparaginase has no onset estimate, because its attributable onsets resolve to three records once matched reports are counted once, below the five-record minimum, whereas its pegylated form is fitted and points toward early onset (α 0.64, 95% CI 0.53–0.89) (Mattano et al., 2000; te Winkel et al., 2011). Voxelotor showed a delayed profile (α 1.43, median 257 days). Glutamine carries the steepest decreasing hazard of any drug fitted (α 0.32, 0.16–0.96), but it rests on seven onset records taking only six distinct values, two of them on the day of therapy start, and its observed median of 98 days lies well above its fitted median of 25 days, so the estimate is weakly identified (Figure 5; Table 5).

### 3.9 The surviving signal set

After all corrections (jaw exclusion, reporter restriction, external validation, empirical calibration, and unmasking, glucocorticoid stratification with misclassification bounding, indication restriction, and temporal signature), each of the 27 intermediate set drugs received one of five final adjudications (Table 2). (1) The glucocorticoid core, comprising prednisone, methylprednisolone, dexamethasone, prednisolone, hydrocortisone, and triamcinolone, is the only class whose association survives every correction and replicates in both external databases. (2) The asparaginase class, asparaginase and pegaspargase, the only residual that combines a large within-indication estimate (asparaginase 7.14, 5.10–10.0; pegaspargase 2.00, 1.26–3.17) with an established mechanism (Mattano et al., 2000; te Winkel et al., 2011; Liu et al., 2016) and an independent external replication (JADER 75.7, 61.4–93.3, from 103 cases). (3) Three further drugs that met the residual criterion but are more economically explained by channeling or co-treatment within their indication and are therefore carried as unresolved rather than as candidate causes, namely testosterone within hypogonadism/androgen deficiency (4.60, 1.96–10.8); arsenic trioxide within acute promyelocytic leukemia (2.72, 1.24–5.99); imiglucerase within Gaucher disease (1.72, 1.26–2.35) (Section 4, Discussion). (4) Indication signals, for which the pre-specified within-indication analysis detected no residual excess over the home disease, namely mycophenolate mofetil, voxelotor, lamivudine, abacavir, dolutegravir, velaglucerase alfa, atazanavir, indinavir, darunavir, tenofovir, stavudine, adefovir, satralizumab, and eliglustat; three of the antiretrovirals do show a residual once the litigation cluster is removed from the HIV stratum, which Section 4, Discussion addresses. (5) Two drugs outside these categories, namely interferon alfa-2b, which was not evaluable because no osteonecrosis case carried the indication, and cholecalciferol, an antiresorptive co-prescription marker. Mycophenolate illustrates the chain of evidence that led to reclassification. Its crude ROR of 2.58 (2.21–3.01) was stable under reporter restriction (2.44, 2.05–2.91) and passed jaw exclusion (JPI 0.12); its GC-unexposed ROR was the weakest of the set (1.52, 1.13–2.05; Firth OR 1.48, 1.27–1.74); reassigning 10% of its 43 GC-negative cases (approximately 5 reports), well within the plausible extent of concomitant medication under-recording, removes that association; within transplant indication reports no residual excess is detected (ROR 0.89, 0.68–1.16; 63 cases among 28,676 reports). The external estimates, Canada 4.05 (1.92–8.55; 7 cases) and JADER 1.78 (1.12–2.84; 18 cases, not meeting the four criteria), are those expected of a marker of the transplant population, whose own association with the endpoint (2.11, 95% CI 1.93–2.31) is of the same size as the drug’s crude signal. Mycophenolate is therefore adjudicated as an indication signal and excluded from the final signal set, which comprises the six glucocorticoids together with asparaginase and pegaspargase (eight drugs). Voxelotor, which the submitted analysis had placed in this set, is excluded on the same principle because it sits exactly at the disease baseline within sickle-cell disease (Table 4).

### 3.10 Machine learning corroboration (XGBoost + SHAP)

As a cross-paradigm consistency check, a gradient-boosted tree classifier (XGBoost) (Chen and Guestrin, 2016) was trained on 144,522 year-stratified reports (24,087 cases with 5 to 1 controls) to predict non-jaw osteonecrosis case status using only report-level demographic and reporting covariates, namely age, sex, reporter occupation, and systemic-GC co-exposure, with no disproportionality output supplied to the learner. The model discriminated cases from controls with an AUROC of 0.765 (± 0.002) and a PR-AUC of 0.470 against a class prevalence baseline of 0.167 (Figure 7). SHAP value attribution (Lundberg and Lee, 2017) ranked the three dominant features as consumer reporting (mean |SHAP| 0.418), lawyer reporting (0.296), and systemic glucocorticoid co-exposure (0.263), whereas professional (HCP) reporting contributed least (0.054). This model-agnostic ranking recovers the two central findings of the study: the signal is carried by non-professional reporting, and glucocorticoid co-exposure dominates the remainder, from the same reports and without being given any disproportionality output. It is therefore a check of internal consistency across two modeling paradigms rather than validation in an independent sample, and it identifies predictive features rather than mechanisms.

**FIGURE 7:**
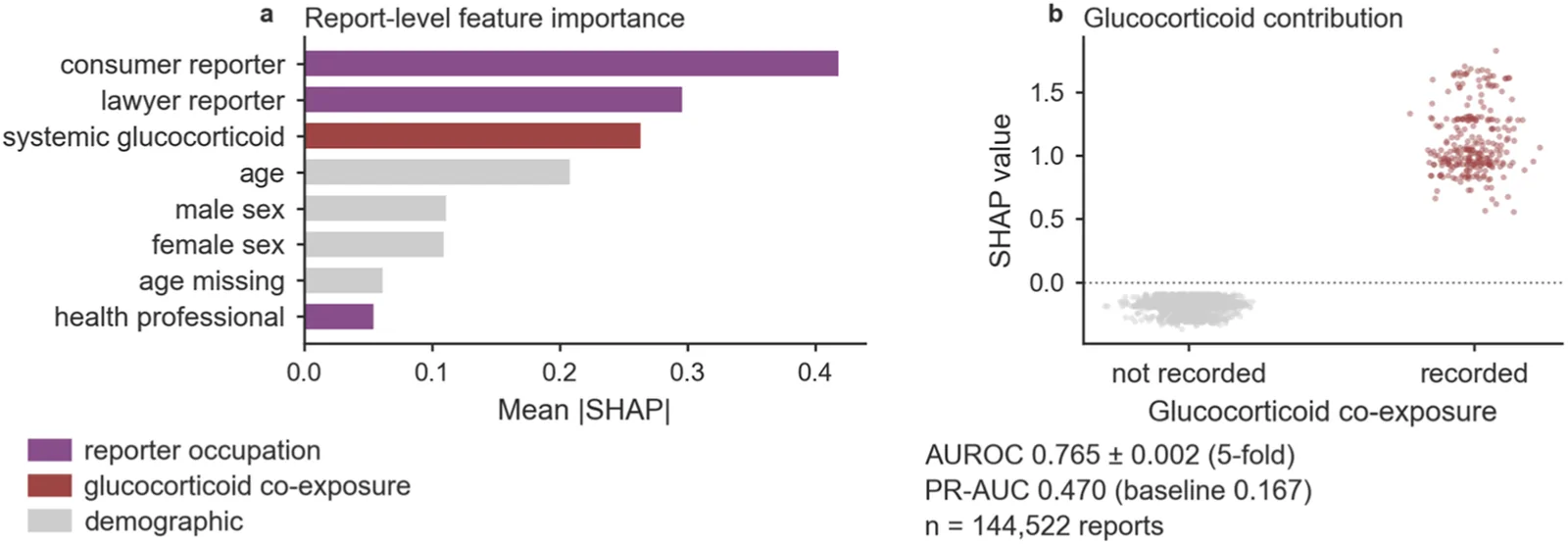
Machine learning corroboration (XGBoost + SHAP). **(a)** Mean absolute SHapley Additive exPlanations (SHAP) value, a model-agnostic measure of feature importance, for an extreme gradient boosting (XGBoost) classifier trained to predict non-jaw osteonecrosis case status from report-level covariates only (age, sex, reporter occupation, systemic glucocorticoid co-exposure; no disproportionality output supplied) on 144,522 year-stratified reports (24,087 cases and five controls per case); violet, reporter occupation features; red, glucocorticoid co-exposure; gray, demographic features. Five-fold cross-validated discrimination is given as the area under the receiver-operating characteristic curve (AUROC) and the area under the precision–recall curve (PR-AUC) against the class prevalence baseline. **(b)** SHAP dependence of systemic glucocorticoid co-exposure (GC), showing that GC exposure shifts predictions toward case status.

## 4 Discussion

Osteonecrosis of the femoral head is a disabling skeletal complication whose drug-related causes are frequently overstated (Mankin and Mankin, 1992; Mont et al., 2015). Its pathophysiology centers on interruption of the femoral head blood supply, in which glucocorticoids promote intramedullary adipocyte hypertrophy, fat embolism, osteocyte apoptosis, and endothelial dysfunction, producing ischemia and eventual subchondral collapse; alcohol, coagulopathy, and trauma are the other dominant predispositions (Mont and Hungerford, 1995; Assouline-Dayan et al., 2002; Zhou et al., 2026; Weinstein, 2012; Kerachian et al., 2009). Against this background, the present study systematically evaluated adverse event reports associated with non-jaw osteonecrosis across 90 quarters of the FAERS database and, critically, corrected two biases that have never before been addressed simultaneously, namely the collapse of the endpoint into a jaw-dominated preferred term and the selective recruitment of reports by litigation.

The results showed that removing jaw misclassification removes the bisphosphonates and denosumab, and removing litigation-driven reporting removes the litigated antiretrovirals; what remains is a glucocorticoid-centered signal plus a small, mechanistically interpretable residue. This reinterprets much of the received full-spectrum account of drug-induced non-jaw osteonecrosis as a consequence of unmodeled reporting structure rather than of pharmacology, with the qualification that disproportionality analysis measures reporting, not risk, and that what these data can show is which associations survive the corrections, not which drugs cause the disease. It should be noted that the two biases act in opposite directions, because jaw contamination inflates the antiresorptive signals while competitive masking from those same inflated signals depresses the ROR of every other drug (Pariente et al., 2007; Maignen et al., 2014). As a result, correcting only one of them cannot produce a stable answer. The conservative explicit-term stratum illustrates how deep the vocabulary problem runs because even the nominally explicit ASEPTIC NECROSIS BONE term carries antiresorptive disproportionality concentrated in the oncology population in which jaw osteonecrosis occurs (Supplementary Table S9). No preferred term list, however curated, substitutes for per-drug site quantification.

H1 was supported, H3 was partly supported, and H2 was refuted in its original form. H1 was supported, in that report-level adjudication plus the descriptive jaw-propensity index separated jaw-contaminated from genuine signals and the index replicated across three databases. H3 was supported in part, in that the Weibull shape parameter distinguished the subacute glucocorticoid signature from the delayed litigation-recruited profile, whereas within the antiresorptive contrast set, only two of the six drugs carry an increasing hazard once each onset record is fitted as the day it names. H2 was refuted in its original form because accounting for glucocorticoid co-exposure did not collapse the non-glucocorticoid survivors into the glucocorticoid stratum; the residual signals instead proved to be disease markers, and it was indication restriction, not glucocorticoid stratification, that resolved them.

Two methodological tools may be applicable beyond this endpoint. The jaw-propensity index operationalizes anatomical site contamination as a per-drug quantity and is computable in any database with a site-specific preferred term. It is descriptive because it is built from the same jaw-site term that defines clinical jaw-centeredness. Its agreement with the six reference antiresorptives is a consistency check, not a validation, and the majority rule threshold of 0.50 is a convention rather than an estimated cut-point. What the two-directional sweep establishes is that the conclusion does not hinge on that convention because the drugs of the final core lie below JPI 0.30 and never change class. In contrast, the threshold moves the antiresorptives with FAERS JPI between 0.50 and 0.65, whose jaw-centeredness is independently supported by their far higher JPI in Canada Vigilance and JADER (alendronate 0.66 and 0.98), by the delayed onset profile of pamidronate and by their oncology populations. The index ordered the drugs the same way in all three databases (r = 0.985 in Canada Vigilance, r = 0.960 in JADER), although the correlation is carried by the separation of two clusters rather than by agreement of individual values, which differ substantially at the boundary (alendronate 0.502 in FAERS against 0.98 in JADER). The cross-database comparison, which sets a signal against the same signal in reporting systems whose recorded solicitation of the drug is far lower, turns an assertion about reporting bias into a set of estimates that can be compared with intervals, without isolating the causal effect of litigation, the tenofovir disoproxil ROR of 157.9 in FAERS falling to 6.33 (5.38–7.45) among health professionals, to 6.24 (2.00–19.4; three cases) in Canada and to 10.5 (6.60–16.8) in JADER. The Canadian estimate is imprecise, and the Japanese estimate is somewhat higher than the FAERS professional estimate, so the external data are directionally consistent with, rather than a confirmation of, the litigation attribution. The most likely reasons for the residual JADER excess are that JADER, in which a consumer qualification appears on 8.4% of reports and a lawyer qualification on 0.02%, behaves as the analog of the FAERS professional stratum rather than of all reporters; that HIV infection itself carries a high osteonecrosis baseline (indication ROR 68.5 in FAERS, 5.62 in JADER), which the whole-corpus estimate does not adjust for; and that tenofovir disoproxil is co-formulated with emtricitabine and efavirenz. Holding the indication constant does not dissolve the contrast, because the litigation lies inside the HIV stratum, so that within HIV-indication reports, the Japanese estimate is 2.75 (1.26–6.00) while the American one is 27.5 (25.2–30.1), 94% of the American HIV-indication cases naming tenofovir disoproxil or a co-formulated partner. A tenofovir–osteonecrosis association of the modest size seen in the Canadian and Japanese data is therefore not excluded; what every such estimate excludes is the FAERS all-reporter magnitude.

The litigation analysis also clearly shows why a second jurisdiction is needed. Excluding reports filed under the lawyer code left 46 of the 47 signals as four-criterion because lawyer-solicited reports enter FAERS mainly through the consumer channel with the occupation field blank or coded as consumer (Rogers et al., 2019; Muñoz and Dal Pan, 2019; Hauben and Harpaz, 2026). The code marks a lower bound of litigation activity (84.5% of tenofovir disoproxil cases) rather than its extent. Restriction to health professional reporters is the more robust screen, and a database whose recorded solicitation of the drug is far lower is the external check on it. For reuse, the cross-jurisdiction test requires (i) a jurisdiction whose recorded solicitation of the drug is demonstrably lower, measured inside the database rather than assumed from the legal record, (ii) an outcome vocabulary and site exclusion that correspond term for term as far as the two coding systems allow, with any residual gap stated, (iii) at least three exposed cases in the comparison database, with the estimate reported with its confidence interval and read as directional evidence, and (iv) comparison against the professional-reporter estimate of the litigated database, because a database in which consumer reporting is a small share resembles that stratum rather than the whole corpus; where the comparison database records indications, restriction to the home indication removes the disease baseline as well. The same reporter filter behaved as expected within a single disease because glutamine, a sickle-cell drug reported only by consumers, was removed, whereas voxelotor, reported more strongly by professionals, was retained.

Competitive masking has a direct clinical reading. When a few drug clusters carry tens of thousands of osteonecrosis reports, the background event rate against which every other drug is compared is inflated, and an ROR of 2.6 for dolutegravir or 4.3 for hydrocortisone is measured against that inflated background rather than against an unbiased one, so its size cannot be read as the size of a small drug effect (Maignen et al., 2014; Wang et al., 2010; Hauben and Maignen, 2017). The unmasking analysis quantifies this, in that removing the antiresorptive and litigated antiretroviral clusters moved the negative control median from 0.16 to 0.38 and rescaled the intermediate set RORs by a factor of approximately 2.35. Additionally, removing the glucocorticoids moved the negative controls to 0.44, and 43 associations that the primary scan could not see crossed the four-criterion thresholds, 84% of them for transplant immunosuppressants, leukemia and myeloma chemotherapy, breast- and prostate cancer therapies, sickle-cell and thalassemia drugs, osteoporosis anabolics, and rheumatology immunomodulators, that is, for the populations in which glucocorticoid exposure, malignancy, or hemoglobinopathy is the plausible cause. Three consequences follow for readers of disproportionality studies of this endpoint. The surviving associations are measured against a comparator that the dominant clusters depress, so removing those clusters raises them; that is a property of the comparator and not a bound on any underlying effect. The absence of a signal for a drug with few osteonecrosis reports is not evidence of safety, because a comparator depressed to this degree leaves little room for a modest association to stand out. The associations revealed by unmasking are, like the residuals of the primary scan, disease markers that require indication-level adjudication before any causal reading.

Mycophenolate retained a GC-independent association (Firth OR 1.48). Its role in solid-organ transplantation, where glucocorticoid exposure carries a high background risk of osteonecrosis, prompted further evaluation. Three analyses informed its interpretation. The association is the most fragile of the set, explained away by misclassification of 10% of its GC-negative cases; no residual excess remains within transplant indication reports (ROR 0.89, 0.68–1.16); and the two external estimates are those of a transplant marker rather than of a drug effect. No residual excess is detected once the transplant population is held constant, which is most consistent with confounding by indication, and mycophenolate is therefore excluded from the final signal set. Spontaneous data cannot distinguish an association that is wholly attributable to the indication from one in which a small direct or potentiating drug effect is also present, and only cohort data can settle that.

The asparaginase signal is the surviving non-glucocorticoid signal with the most plausible direct mechanism. Asparaginase depletes asparagine and reduces hepatic synthesis of antithrombin, protein C, protein S, and plasminogen, producing a hypercoagulable state. It also inhibits lipoprotein lipase, producing hypertriglyceridemia. Both are established osteonecrosis risk factors, and in the mouse, asparaginase potentiates glucocorticoid-induced osteonecrosis by increasing dexamethasone exposure (Liu et al., 2016). In acute lymphoblastic leukemia, where asparaginase is co-administered with dexamethasone or prednisone, symptomatic osteonecrosis affects roughly 5%–17% of adolescents and young adults. Randomized protocol comparisons have shown that the schedule of dexamethasone and the intensity of asparaginase-containing post-induction therapy change its incidence (Mattano et al., 2000; te Winkel et al., 2011; Mattano et al., 2012; Mattano et al., 2024; Valtis et al., 2022).

In the present data, the asparaginase ROR remained elevated within leukemia-indication reports (7.14, 5.10–10.0) after holding the leukemia baseline constant. Its glucocorticoid-exposed stratum ROR was 10.3. Its onset profile carries no estimate because too few onset records remain after matching potential duplicate reports, and the signal replicated in JADER with ROR 75.7 (61.4–93.3) from 103 cases, the strongest external replication of any non-glucocorticoid drug. Its pegylated form, pegaspargase, which has replaced native asparaginase in most current protocols, showed the same direction with a smaller estimate (2.00, 1.26–3.17), consistent with a class effect whose magnitude depends on era, protocol intensity, and cumulative glucocorticoid exposure rather than on the formulation itself. The clinical implication is that hip and knee pain in adolescents receiving asparaginase-containing regimens should prompt imaging, and that trials modifying asparaginase intensity should record osteonecrosis as a secondary outcome; disentangling the asparaginase-specific effect from the glucocorticoid component of the regimen still requires cohort data rather than disproportionality.

The disease-marker signals are mechanistically interpretable, and the sickle-cell case shows why the indication comparator must be built correctly. Gaucher disease and sickle-cell anemia are themselves osteonecrosis-producing, as glucocerebroside-laden macrophages infiltrate the marrow and compromise femoral head vascularity in Gaucher disease, and recurrent vaso-occlusion of the femoral head microcirculation drives ischemic necrosis in sickle-cell disease (Milner et al., 1991; Wenstrup et al., 2002). Both diseases are strongly associated with the endpoint in these data (indication ROR 19.8 and 6.08). Voxelotor is the clearest example. Across the whole corpus, it looks like a substantial signal (ROR 5.06). It is reported more strongly by health professionals (23.2, 18.6–29.0) than by all reporters and is almost never lawyer-sourced (0.8%), so neither jaw contamination nor litigation explains it. A mechanism is even conceivable because voxelotor increases the oxygen affinity of hemoglobin S to inhibit polymerization (Vichinsky et al., 2019; Howard et al., 2021), and a left-shifted oxygen-dissociation curve could, in principle, reduce oxygen unloading in the poorly perfused femoral head marrow. Within sickle-cell disease, however, the signal disappears (0.97, 0.71–1.32) because voxelotor accounts for 64% of the reports and 64% of the osteonecrosis cases of that stratum, which is what a drug that simply marks the treated population looks like. The submitted version of this analysis, computed on an indication table that had been restricted upstream to candidate osteonecrosis reports, had placed voxelotor among the residual signals; rebuilding the indication table for the whole corpus reverses that call, and we report the corrected result. The Gaucher-directed therapies behave the same way, with a gradient across the enzyme replacements and the substrate-reduction agent eliglustat (imiglucerase 1.72 (1.26–2.35), velaglucerase alfa 0.85 (0.58–1.25), and eliglustat 0.27 (0.14–0.53)), which could reflect differences in disease severity, treatment duration, or prescribing pattern, none of which is recorded in these data, rather than a pharmacological difference. Voxelotor was voluntarily withdrawn from all markets in September 2024, within our study window, after clinical data showed an unfavorable benefit–risk balance (Pfizer Inc., 2024; U.S. Food and Drug Administration, 2024; Kim and Prasad, 2025). The concerns involved an imbalance in vaso-occlusive crises and fatal events; an independent FAERS analysis also flagged osteonecrosis among its unlabeled events (Lin et al., 2025). Our analysis shows that such a flag is expected for any sickle-cell drug from the disease baseline alone, which is precisely why disproportionality signals in severely affected populations should not be read without an indication-restricted comparison.

The non-litigated antiretrovirals are harder to classify because HIV infection itself is an established osteonecrosis risk factor (indication ROR 68.5 in FAERS and 5.62 in JADER) (Miller et al., 2002; Glesby et al., 2001; Morse et al., 2007; Borges et al., 2017; Bahoravitch et al., 2023). All eight within-HIV-indication reports fell to or below the HIV baseline, several of them far below it, which is what a stratum dominated by the litigation cluster produces (within-HIV ROR 0.06–1.34, every lower 95% bound ≤ 1). They are adjudicated as indication signals; that is, no residual excess over the HIV baseline was detected for them. Because 94% of its osteonecrosis cases name tenofovir disoproxil or a co-formulated partner, we repeated the restriction in the cleaner stratum that remains once those reports are removed. Most of the antiretrovirals stay at the disease baseline there as well. Only atazanavir (1.62, 1.08–2.44), indinavir (12.2, 7.49–19.9), and stavudine (3.83, 2.15–6.84) retained a lower bound above 1, and those are precisely the early protease-inhibitor and thymidine-analog agents of the era in which HIV-associated osteonecrosis incidence peaked. We therefore keep the pre-specified primary analysis verdict as an indication signal, while noting that these three residuals are sensitive to the composition of the comparator. Confounding by treatment era, disease stage, and regimen-associated dyslipidemia remains the more economical reading, but these data do not establish it. None of the three is externally testable, and spontaneous data cannot separate a small direct effect from the treatment era in which it would have occurred. Litigation does not explain these three, whose reports kept their health professional signals and whose onset profiles (α 0.93, 0.79 and 1.19) lack the uniformly delayed signature of the litigated antiretrovirals (1.30–1.45), although stavudine approaches it. The other retained antiretrovirals are likewise unlike the litigated ones (α 0.72–0.99 for lamivudine, dolutegravir, and darunavir). Of the remaining low-count signals, adefovir and satralizumab showed no residual excess over the home-disease baseline and interferon alfa-2b was not evaluable, whereas testosterone and arsenic trioxide remained elevated within their home indication. Both of the latter sit in strata that carry their own osteonecrosis risk because patients with acute promyelocytic leukemia receive high-dose glucocorticoids for differentiation syndrome, and hypogonadism is itself a consequence of chronic illness, opioid therapy, and prior glucocorticoid exposure, so testosterone reports select a population already at risk. Only 35% of its osteonecrosis cases carry the indication at all. Neither is externally testable; each rests on a dozen to forty-odd within-indication cases, and neither is advanced as a candidate cause. The same reasoning applies to imiglucerase, whose within-Gaucher estimate (1.72, 1.26–2.35) exceeds those of the other enzyme therapies. As the oldest and most widely used agent, it is plausibly given to the longest and most severely treated patients, but neither treatment duration nor disease severity is recorded in these data, so the excess is unresolved.

The external replication should be read with the structural differences between the three databases in mind (Supplementary Table S6). FAERS accepts consumer and lawyer reports and contains the litigation; Canada Vigilance accepts consumer reports (21% of its reports) and has a lawyer channel of its own (0.3%), but its recorded lawyer channel carries almost none of this endpoint, and it covers a shorter period (to November 2024); JADER is forwarded largely by marketing-authorization holders and medical institutions, carries a consumer qualification on 8.4% of reports and a lawyer qualification on 0.02%, is coded in MedDRA/J, and includes prednisolone where FAERS includes prednisone. These differences can produce both false replications (a shared reporting culture) and false non-replications (a drug rarely used in one country, such as prednisone in Japan or voxelotor outside the United States). They also mean that effect sizes are not expected to agree, and the glucocorticoid class ROR of 54.4 in JADER against 10.3 in Canada and 3.0–12.4 per drug in FAERS reflects the different background composition of each corpus rather than a different biology. Concordance was therefore judged on direction and on four-criterion status, and only for drugs with at least three external cases.

This study has several limitations. First, the jaw-propensity index is descriptive, and its agreement with reference antiresorptives is circular because both depend on the same jaw-site term. It flags plausible contamination without quantifying its extent or establishing an anatomical site in reports without an explicit location. Second, spontaneous reports remain susceptible to under-reporting, notoriety bias, and competitive masking (Pariente et al., 2007; Maignen et al., 2014); the unmasking analysis provides only a limited assessment of masking. Third, incomplete concomitant medication recording leaves residual glucocorticoid exposure misclassification. Sensitivity analyses bounded, but could not eliminate, this uncertainty. Fourth, sparse external cases limited testing to 5 of 27 intermediate set drugs in Canada Vigilance and 13 of 27 in JADER, reducing power to replicate weaker signals. Fifth, indication matching by MedDRA Preferred Term substrings may miss indications or misclassify them in either direction and cannot link indications to individual drugs in multidrug reports. Comparisons have limited power when one drug dominates its indication stratum, so an undetected residual excess does not exclude a drug contribution. External case definitions are database-specific approximations of the adjudicated scheme. These adjudications are not clinical diagnoses. Sixth, empirical null calibration addresses global bias but cannot model drug-specific litigation bias, and cross-database comparisons cannot isolate litigation’s causal effect. Because solicited reports can enter through consumer channels, lawyer coding provides only a lower bound on solicitation; low lawyer-coded reporting does not establish low recruitment. Seventh, drug-attributable onset data were available for only 27.1% of cases. Imprecise dates and sparse samples leave Weibull shape parameters uncertain; drugs with fewer than five onset records after matching potential duplicate reports were not fitted. Eighth, the primary scan pooled all quarters and corrected for 432 tests, but secondary analyses, including stratification, indication restriction, and external replication, were not adjusted for multiplicity. Their concordance is exploratory, not confirmatory. Prospective studies are needed to evaluate these associations.

## 5 Conclusion

Once jaw misclassification, litigation-driven reporting, and confounding by indication are accounted for, the reported drug spectrum of non-jaw osteonecrosis is glucocorticoid-centered, and the asparaginase class is the best-supported residual, carrying a specific mechanism and replicating in JADER, although spontaneous reports cannot establish that it is causal. Three additional drugs met the residual criterion and remain unresolved. The widely cited bisphosphonate and antiretroviral osteonecrosis signals are consistent with, respectively, jaw ONJ misclassified into the osteonecrosis preferred term and solicitation of reports during mass tort litigation. Both were reproducibly attenuated by report-level adjudication, reporter restriction, and comparison with two databases in which the recorded solicitation of these drugs is far lower. The jaw-propensity index, used as a descriptive contamination index, and the cross-jurisdiction litigation test are reusable for any litigated, misclassification-prone pharmacovigilance endpoint. Future case definitions for this endpoint should operate at the report level, exclude oral site co-events, quantify per-drug jaw propensity, and be validated against a database in which the recorded solicitation of the drug is measurably lower.

## Data Availability

The original contributions presented in the study are included in the article/Supplementary Material; further inquiries can be directed to the corresponding authors.
