# Supplementary material for: Glucocorticoid-centered drug spectrum of non-jaw osteonecrosis, a FAERS pharmacovigilance study with independent validation in Canada Vigilance and JADER
Source: Front Pharmacol. 2026 Oct 21;17:1977191. doi: 10.3389/fphar.2026.1977191 (PMC13731382; doi:10.3389/fphar.2026.1977191)
Supplement: Supplementary file 1 [file Supplementaryfile1.docx]

**Supplementary Material for Glucocorticoid-centred drug spectrum of non-jaw osteonecrosis, a FAERS pharmacovigilance study with independent validation in Canada Vigilance and JADER**

This supplement provides the methods tables, the complete per-drug results that underpin every number reported in the main text, and the additional sensitivity analyses added at revision (marked in red). Each table corresponds to a reproducible analysis step of the modular pipeline (src/) and to a CSV in out/tab/; all tables were regenerated from a single end-to-end re-execution of the pipeline in September 2026. Supplementary tables and figures are numbered in the order in which the main text first cites them.

**Supplementary Table S1. Disproportionality analysis with the 2 × 2 table and signal-detection criteria**

**S1a. Four-fold table of disproportionality analysis.**

|  | **Target adverse event** | **All other adverse events** | **Total** |
| --- | --- | --- | --- |
| Target drug | a | b | a + b |
| All other drugs | c | d | c + d |
| Total | a + c | b + d | a + b + c + d |

a is the number of reports of the target adverse event associated with the target drug; b the number of reports of all other adverse events associated with the target drug; c the number of reports of the target adverse event associated with all other drugs; and d the number of reports of all other adverse events associated with all other drugs.

**S1b. Algorithms and signal-detection criteria.**

| **Algorithm** | **Formula** | **Signal criterion** |
| --- | --- | --- |
| ROR | ROR = (a·d)/(b·c); 95% CI = exp[ln(ROR) ± 1.96·√(1/a + 1/b + 1/c + 1/d)] | 95% CI lower bound > 1, a ≥ 3 |
| PRR | PRR = (a/(a+b)) / (c/(c+d)); χ² = (a·d − b·c)²·(a+b+c+d) / [(a+b)(c+d)(a+c)(b+d)] | PRR ≥ 2, χ² ≥ 4, a ≥ 3 |
| MGPS | E = (a+b)(a+c)/(a+b+c+d); posterior λ ~ Gamma(shape = a + 0.5, rate = E + 0.5); EBGM = posterior mean = (a+0.5)/(E+0.5); EBGM05 = exact 5th percentile of that Gamma posterior | EBGM05 > 2 |
| BCPNN | E = (a+b)(a+c)/(a+b+c+d); IC = log₂[(a+0.5)/(E+0.5)]; IC025 = IC − 1.96·√(1/(a+0.5) + 1/(E+0.5))/ln(2) | IC025 > 0 |

E is the expected count under independence. EBGM and IC apply a one-component gamma-Poisson shrinkage in the spirit of DuMouchel, adding 0.5 to the observed and expected counts; this is the single-component form, not the two-component empirical-Bayes mixture of the original MGPS, so EBGM here is the mean of a Gamma(a + 0.5, E + 0.5) posterior and EBGM05 its exact fifth percentile, obtained from the Gamma distribution function rather than from a lognormal approximation to it. A lognormal approximation to that quantile, which earlier versions of this analysis used, is anti-conservative for drugs with few cases, which is where the EBGM05 = 2 threshold operates, and is not used here. IC025 retains the usual normal approximation on the log2 scale. A drug was called a robust signal only when all four criteria were met simultaneously (pre-specified in the statistical analysis plan). The Fisher exact test with Bonferroni correction over the 432 ingredients tested (a ≥ 3) was applied to the 2 × 2 table.

Rationale for the four-criterion rule. The four measures have complementary weaknesses, in that ROR and PRR are frequentist ratios that are unstable when a is small, whereas EBGM05 and IC025 shrink small counts toward the null and are conservative for rare drugs. Requiring all four to be met is a consensus filter that trades sensitivity for specificity; in a scan of 432 ingredients, where false-positive signals are the dominant risk, this is the appropriate direction of the trade-off.

**Supplementary Table S2. Jaw-propensity index (JPI), two-directional threshold sensitivity and knowledge-based consistency check**

S2a. Two-directional threshold sweep. At each threshold the intermediate survivor set was rebuilt from the same two pre-specified filters (JPI below the threshold and reporter-restriction pass) and compared with the primary set (threshold 0.50, n = 27). The leave column lists drugs that drop out of the set and the enter column lists drugs that would be admitted. Sensitivity, specificity and the Youden J index are computed against the knowledge-based reference set of six jaw-centred drugs (five bisphosphonates plus denosumab) versus the systemic glucocorticoids of the signal set; because the JPI is computed from the same jaw-site preferred term that defines clinical jaw-centredness, this is a consistency check rather than an independent validation. The final core (six glucocorticoids, asparaginase, pegaspargase) never changes class at any threshold. See also Supplementary Figure S1.

| **Threshold** | **Sensitivity** | **Specificity** | **Youden J** | **Survivors (n)** | **Leave (n)** | **Leave** | **Enter (n)** | **Enter** |
| --- | --- | --- | --- | --- | --- | --- | --- | --- |
| 0.30 | 1.00 | 1.00 | 1.00 | 25 | 2 | Arsenic Trioxide; Cholecalciferol | 0 | n.a. |
| 0.35 | 1.00 | 1.00 | 1.00 | 25 | 2 | Arsenic Trioxide; Cholecalciferol | 0 | n.a. |
| 0.40 | 1.00 | 1.00 | 1.00 | 25 | 2 | Arsenic Trioxide; Cholecalciferol | 0 | n.a. |
| 0.45 | 1.00 | 1.00 | 1.00 | 26 | 1 | Cholecalciferol | 0 | n.a. |
| 0.50 | 1.00 | 1.00 | 1.00 | 27 | 0 | n.a. | 0 | n.a. |
| 0.55 | 0.83 | 1.00 | 0.83 | 28 | 0 | n.a. | 1 | Alendronate |
| 0.60 | 0.67 | 1.00 | 0.67 | 29 | 0 | n.a. | 2 | Alendronate; Pamidronate |
| 0.65 | 0.50 | 1.00 | 0.50 | 30 | 0 | n.a. | 3 | Alendronate; Ibandronate; Pamidronate |
| 0.70 | 0.50 | 1.00 | 0.50 | 30 | 0 | n.a. | 3 | Alendronate; Ibandronate; Pamidronate |

S2b. Per-drug JPI for the knowledge-based reference set, with the JPI recomputed in Canada Vigilance and JADER where those databases contribute at least three osteonecrosis-family cases for the drug.

| **Ingredient** | **a (FAERS)** | **n jaw** | **JPI (FAERS)** | **Reference class** | **JPI (Canada)** | **JPI (JADER)** |
| --- | --- | --- | --- | --- | --- | --- |
| Denosumab | 481 | 8,005 | 0.943 | jaw | 0.928 | 0.996 |
| Risedronate | 60 | 212 | 0.779 | jaw | 0.930 | 0.989 |
| Zoledronic Acid | 2,945 | 7,312 | 0.713 | jaw | 0.740 | 0.991 |
| Ibandronate | 266 | 491 | 0.649 | jaw | n.a. | 0.981 |
| Pamidronate | 684 | 844 | 0.554 | jaw | 0.667 | 0.969 |
| Alendronate | 2,457 | 2,477 | 0.502 | jaw | 0.661 | 0.981 |
| Prednisolone | 336 | 128 | 0.276 | non-jaw | n.a. | 0.229 |
| Dexamethasone | 312 | 96 | 0.235 | non-jaw | 0.185 | 0.227 |
| Prednisone | 392 | 73 | 0.157 | non-jaw | 0.138 | n.a. |
| Methylprednisolone | 377 | 23 | 0.058 | non-jaw | 0.000 | 0.042 |
| Hydrocortisone | 51 | 2 | 0.038 | non-jaw | 0.000 | 0.021 |
| Triamcinolone | 59 | 0 | 0.000 | non-jaw | n.a. | 0.000 |

**Supplementary Table S3. Adversarial glucocorticoid-misclassification tipping point for every evaluable drug**

For each drug with at least ten osteonecrosis cases in the glucocorticoid-unexposed stratum, the tipping fraction f* is the smallest share of its GC-negative cases that, if reassigned to the GC-exposed stratum (misclassification concentrated entirely in the drug-exposed case cell), brings the lower 95% bound of the GC-negative ROR to 1. Drugs whose lower bound is still above 1 at f = 0.99 are reported as greater than 0.99, and cases reassigned is the corresponding number of reports. Calls are graded fragile below 0.30, moderate 0.30 to 0.59 and robust 0.60 or above. See also Supplementary Figure S2.

| **Ingredient** | **Cases (all)** | **Cases GC+** | **Cases GC−** | **GC− ROR** | **GC− ROR lower bound** | **Tipping fraction** | **Cases reassigned** | **Band** |
| --- | --- | --- | --- | --- | --- | --- | --- | --- |
| Mycophenolate Mofetil | 159 | 116 | 43 | 1.52 | 1.13 | 0.10 | ≈5 | fragile |
| Darunavir | 21 | 3 | 18 | 3.24 | 2.04 | 0.44 | ≈8 | moderate |
| Dolutegravir | 45 | 4 | 41 | 2.86 | 2.11 | 0.47 | ≈20 | moderate |
| Tenofovir | 19 | 0 | 19 | 3.83 | 2.44 | 0.51 | ≈10 | moderate |
| Interferon alfa-2b | 14 | 1 | 13 | 4.33 | 2.51 | 0.51 | ≈7 | moderate |
| Nevirapine | 29 | 2 | 27 | 4.25 | 2.91 | 0.58 | ≈16 | moderate |
| Testosterone | 126 | 1 | 125 | 3.21 | 2.69 | 0.59 | ≈74 | moderate |
| Denosumab | 481 | 39 | 442 | 2.85 | 2.60 | 0.60 | ≈266 | robust |
| Abacavir | 46 | 1 | 45 | 4.33 | 3.23 | 0.63 | ≈29 | robust |
| Lamivudine | 102 | 3 | 99 | 3.99 | 3.27 | 0.66 | ≈66 | robust |
| Zidovudine | 35 | 1 | 34 | 5.38 | 3.84 | 0.67 | ≈23 | robust |
| Triamcinolone | 59 | 34 | 25 | 5.81 | 3.92 | 0.67 | ≈17 | robust |
| Glutamine | 22 | 0 | 22 | 6.66 | 4.38 | 0.69 | ≈16 | robust |
| Adefovir | 16 | 1 | 15 | 8.81 | 5.30 | 0.71 | ≈11 | robust |
| Atazanavir | 33 | 2 | 31 | 7.66 | 5.38 | 0.74 | ≈23 | robust |
| Arsenic Trioxide | 14 | 1 | 13 | 11.35 | 6.57 | 0.75 | ≈10 | robust |
| Voxelotor | 128 | 1 | 127 | 5.83 | 4.89 | 0.76 | ≈97 | robust |
| Lopinavir | 52 | 2 | 50 | 8.60 | 6.51 | 0.79 | ≈40 | robust |
| Risedronate | 60 | 7 | 53 | 9.55 | 7.28 | 0.81 | ≈43 | robust |
| Nelfinavir | 11 | 0 | 11 | 19.12 | 10.53 | 0.81 | ≈9 | robust |
| Taliglucerase Alfa | 10 | 0 | 10 | 21.22 | 11.34 | 0.81 | ≈9 | robust |
| Rilpivirine | 137 | 2 | 135 | 8.59 | 7.24 | 0.83 | ≈113 | robust |
| Stavudine | 19 | 1 | 18 | 22.30 | 13.98 | 0.86 | ≈16 | robust |
| Velaglucerase Alfa | 36 | 1 | 35 | 19.00 | 13.60 | 0.87 | ≈31 | robust |
| Ibandronate | 266 | 21 | 245 | 12.35 | 10.88 | 0.89 | ≈219 | robust |
| Cobicistat | 155 | 8 | 147 | 20.19 | 17.14 | 0.92 | ≈136 | robust |
| Elvitegravir | 152 | 8 | 144 | 26.45 | 22.40 | 0.93 | ≈134 | robust |
| Indinavir | 22 | 3 | 19 | 62.16 | 39.09 | 0.93 | ≈18 | robust |
| Imiglucerase | 118 | 1 | 117 | 28.43 | 23.65 | 0.94 | ≈110 | robust |
| Cholecalciferol | 71 | 7 | 64 | 36.25 | 28.24 | 0.94 | ≈61 | robust |
| Efavirenz | 588 | 20 | 568 | 45.06 | 41.38 | 0.97 | ≈551 | robust |
| Zoledronic Acid | 2,945 | 344 | 2,601 | 55.61 | 53.32 | 0.98 | ≈2549 | robust |
| Emtricitabine | 2,579 | 86 | 2,493 | 53.57 | 51.32 | 0.98 | ≈2444 | robust |
| Alendronate | 2,457 | 166 | 2,291 | 86.17 | 82.38 | 0.99 | ≈2269 | robust |
| Tenofovir Disoproxil | 7,027 | 112 | 6,915 | 202.88 | 196.76 | not reached at f = 0.99 | n.a. (censored) | robust |
| Pamidronate | 684 | 128 | 556 | 233.34 | 212.57 | not reached at f = 0.99 | n.a. (censored) | robust |

**Supplementary Table S4. The 34 negative controls for empirical-null calibration**

Drugs with no known or plausible non-jaw osteonecrosis mechanism (antihistamines, proton-pump inhibitors, antihypertensives and diuretics, oral antidiabetics, statins, selective serotonin reuptake inhibitors, levothyroxine, acetaminophen, gabapentinoids, salbutamol, non-steroidal anti-inflammatory drugs and montelukast), used to estimate the systematic-error null N(μ, σ) on the natural-log ROR scale. Median ROR = 0.16; all 34 ROR < 1, confirming competitive masking. Anticoagulants and vitamin D were excluded a priori.

| **Ingredient** | **a** | **ROR** | **ln ROR** | **SE** |
| --- | --- | --- | --- | --- |
| Loratadine | 0 | 0.012 | -4.40 | 1.41 |
| Cetirizine | 3 | 0.088 | -2.44 | 0.53 |
| Fexofenadine | 1 | 0.045 | -3.10 | 0.82 |
| Levocetirizine | 0 | 0.044 | -3.11 | 1.41 |
| Diphenhydramine | 1 | 0.070 | -2.66 | 0.82 |
| Omeprazole | 15 | 0.345 | -1.06 | 0.25 |
| Pantoprazole | 15 | 0.405 | -0.90 | 0.25 |
| Esomeprazole | 32 | 0.397 | -0.92 | 0.18 |
| Amlodipine | 13 | 0.178 | -1.73 | 0.27 |
| Lisinopril | 26 | 0.731 | -0.31 | 0.19 |
| Losartan | 3 | 0.152 | -1.88 | 0.53 |
| Valsartan | 23 | 0.135 | -2.00 | 0.21 |
| Ramipril | 3 | 0.158 | -1.84 | 0.53 |
| Metoprolol | 2 | 0.088 | -2.43 | 0.63 |
| Hydrochlorothiazide | 11 | 0.273 | -1.30 | 0.30 |
| Furosemide | 3 | 0.125 | -2.08 | 0.53 |
| Metformin | 16 | 0.155 | -1.86 | 0.25 |
| Glimepiride | 0 | 0.131 | -2.03 | 1.41 |
| Sitagliptin | 5 | 0.132 | -2.02 | 0.43 |
| Atorvastatin | 21 | 0.202 | -1.60 | 0.22 |
| Simvastatin | 8 | 0.231 | -1.47 | 0.34 |
| Rosuvastatin | 9 | 0.176 | -1.74 | 0.32 |
| Sertraline | 16 | 0.253 | -1.37 | 0.25 |
| Citalopram | 5 | 0.180 | -1.72 | 0.43 |
| Escitalopram | 4 | 0.164 | -1.81 | 0.47 |
| Fluoxetine | 2 | 0.078 | -2.55 | 0.63 |
| Levothyroxine | 9 | 0.145 | -1.93 | 0.32 |
| Acetaminophen | 6 | 0.028 | -3.57 | 0.39 |
| Gabapentin | 17 | 0.215 | -1.54 | 0.24 |
| Pregabalin | 30 | 0.221 | -1.51 | 0.18 |
| Albuterol | 21 | 0.195 | -1.63 | 0.22 |
| Naproxen | 4 | 0.057 | -2.87 | 0.47 |
| Ibuprofen | 24 | 0.231 | -1.46 | 0.20 |
| Montelukast | 23 | 0.887 | -0.12 | 0.21 |

**Supplementary Table S5. Unmasking sensitivity analysis**

S5a. The primary scan was recomputed after removing from the analysis universe every report in which a masking-cluster drug was a suspect drug, first the six antiresorptives and the six litigated antiretrovirals (stage 1) and then additionally the seven drugs of the systemic glucocorticoid masking set (stage 2). See also Supplementary Figure S3.

| **Stage** | **Reports removed** | **Cases removed** | **Universe** | **Ingredients tested** | **Four-criterion signals** | **Primary signals retained** | **New signals (n)** | **New signals** | **Negative-control median ROR** | **Negative controls < 1** |
| --- | --- | --- | --- | --- | --- | --- | --- | --- | --- | --- |
| primary | 0 | 0 | 20,690,895 | 432 | 47 | 47 / 47 | 0 | n.a. | 0.16 | 100% |
| stage1 no antiresorptive no litigatedARV | 383,790 | 13,986 | 20,307,105 | 416 | 76 | 33 / 35 | 43 | Anastrozole; Azathioprine; Belumosudil; Betamethasone; Bevacizumab; Blinatumomab; Crizanlizumab; Cyclophosphamide; Cyclosporine; Cytarabine; Deferasirox; Doxorubicin; Duragesic-100; Etoposide; Exemestane; Fludarabine; Fulvestrant; Goserelin; Hydroxychloroquine; Hydroxyurea; Imatinib; Interferon Beta-1A; Isopropyl Alcohol; Leflunomide; Letrozole; Lymphocyte Immune Globulin, Anti-Thymocyte Globulin; Mabthera; Melphalan; Methotrexate; Miglustat; Raloxifene; Raltegravir; Ritonavir; Rituximab; Romosozumab; Sirolimus; Somatropin; Tacrolimus; Taxotere; Thalidomide; Tretinoin; Upadacitinib; Vincristine | 0.38 | 94% |
| stage2 also no glucocorticoid | 571,815 | 15,532 | 20,119,080 | 409 | 72 | 27 / 29 | 45 | Alectinib; Anastrozole; Azathioprine; Belumosudil; Bevacizumab; Blinatumomab; Crizanlizumab; Cyclophosphamide; Cyclosporine; Cytarabine; Deferasirox; Doxorubicin; Duragesic-100; Etoposide; Exemestane; Ferric Carboxymaltose; Fludarabine; Fulvestrant; Goserelin; Hydroxychloroquine; Hydroxyurea; Imatinib; Interferon Beta-1A; Isopropyl Alcohol; Leflunomide; Letrozole; Lymphocyte Immune Globulin, Anti-Thymocyte Globulin; Mabthera; Melphalan; Methotrexate; Miglustat; Raloxifene; Raltegravir; Ritonavir; Rituximab; Romosozumab; Sirolimus; Somatropin; Tacrolimus; Taxotere; Teriparatide; Thalidomide; Tretinoin; Upadacitinib; Vincristine | 0.44 | 88% |

S5b. Per-drug ROR under the primary universe and each unmasking stage, covering the 27-drug intermediate set, the 20 primary signals removed by the jaw or reporter filters, the 34 negative controls, and every ingredient that newly meets all four criteria after unmasking. For drugs outside the removed clusters unmasking rescales the ROR by an almost constant factor, so ranks are preserved and only threshold crossings change. Drugs that belong to a removed cluster are not evaluable in that stage. For the newly unmasked associations the last column gives the population in which the drug is used; these associations were not carried through the adjudication pipeline and are hypothesis-generating. Three entries are unmapped brand names (Mabthera = rituximab; Taxotere = docetaxel; Duragesic-100 = fentanyl).

| **Ingredient** | **Group** | **ROR primary** | **ROR stage 1** | **ROR stage 2** | **Ratio s1/primary** | **Ratio s2/primary** | **Four criteria (primary)** | **(stage 1)** | **(stage 2)** | **Population / class** |
| --- | --- | --- | --- | --- | --- | --- | --- | --- | --- | --- |
| Montelukast | negative control | 0.868 | 2.036 | 2.382 | 2.35 | 2.74 | no | no | no | n.a. |
| Lisinopril | negative control | 0.717 | 1.681 | 1.968 | 2.35 | 2.75 | no | no | no | n.a. |
| Pantoprazole | negative control | 0.392 | 0.918 | 1.074 | 2.34 | 2.74 | no | no | no | n.a. |
| Esomeprazole | negative control | 0.391 | 0.918 | 1.075 | 2.35 | 2.75 | no | no | no | n.a. |
| Omeprazole | negative control | 0.334 | 0.783 | 0.916 | 2.34 | 2.74 | no | no | no | n.a. |
| Hydrochlorothiazide | negative control | 0.262 | 0.613 | 0.717 | 2.34 | 2.74 | no | no | no | n.a. |
| Sertraline | negative control | 0.245 | 0.575 | 0.673 | 2.34 | 2.74 | no | no | no | n.a. |
| Ibuprofen | negative control | 0.227 | 0.531 | 0.622 | 2.34 | 2.74 | no | no | no | n.a. |
| Pregabalin | negative control | 0.218 | 0.511 | 0.598 | 2.35 | 2.75 | no | no | no | n.a. |
| Simvastatin | negative control | 0.217 | 0.509 | 0.596 | 2.34 | 2.74 | no | no | no | n.a. |
| Gabapentin | negative control | 0.208 | 0.489 | 0.572 | 2.34 | 2.74 | no | no | no | n.a. |
| Atorvastatin | negative control | 0.197 | 0.463 | 0.542 | 2.34 | 2.74 | no | no | no | n.a. |
| Albuterol | negative control | 0.191 | 0.447 | 0.523 | 2.34 | 2.74 | no | no | no | n.a. |
| Amlodipine | negative control | 0.171 | 0.402 | 0.470 | 2.34 | 2.74 | no | no | no | n.a. |
| Rosuvastatin | negative control | 0.167 | 0.390 | 0.457 | 2.34 | 2.74 | no | no | no | n.a. |
| Citalopram | negative control | 0.163 | 0.382 | 0.447 | 2.34 | 2.74 | no | no | no | n.a. |
| Metformin | negative control | 0.150 | 0.353 | 0.413 | 2.34 | 2.74 | no | no | no | n.a. |
| Escitalopram | negative control | 0.145 | 0.341 | 0.398 | 2.34 | 2.74 | no | no | no | n.a. |
| Levothyroxine | negative control | 0.137 | 0.321 | 0.375 | 2.34 | 2.74 | no | no | no | n.a. |
| Ramipril | negative control | 0.136 | 0.318 | 0.372 | 2.34 | 2.74 | no | no | no | n.a. |
| Valsartan | negative control | 0.132 | 0.309 | 0.362 | 2.34 | 2.74 | no | no | no | n.a. |
| Glimepiride | negative control | 0.131 | 0.308 | 0.360 | 2.34 | 2.74 | no | no | no | n.a. |
| Losartan | negative control | 0.130 | 0.305 | 0.357 | 2.34 | 2.74 | no | no | no | n.a. |
| Sitagliptin | negative control | 0.120 | 0.282 | 0.330 | 2.34 | 2.74 | no | no | no | n.a. |
| Furosemide | negative control | 0.107 | 0.251 | 0.293 | 2.34 | 2.74 | no | no | no | n.a. |
| Metoprolol | negative control | 0.088 | 0.206 | 0.242 | 2.34 | 2.74 | no | no | no | n.a. |
| Fluoxetine | negative control | 0.078 | 0.182 | 0.213 | 2.34 | 2.74 | no | no | no | n.a. |
| Cetirizine | negative control | 0.075 | 0.176 | 0.206 | 2.34 | 2.74 | no | no | no | n.a. |
| Diphenhydramine | negative control | 0.070 | 0.164 | 0.192 | 2.34 | 2.74 | no | no | no | n.a. |
| Naproxen | negative control | 0.050 | 0.118 | 0.138 | 2.34 | 2.74 | no | no | no | n.a. |
| Fexofenadine | negative control | 0.045 | 0.106 | 0.124 | 2.34 | 2.74 | no | no | no | n.a. |
| Levocetirizine | negative control | 0.044 | 0.104 | 0.122 | 2.34 | 2.74 | no | no | no | n.a. |
| Acetaminophen | negative control | 0.026 | 0.061 | 0.071 | 2.34 | 2.74 | no | no | no | n.a. |
| Loratadine | negative control | 0.012 | 0.029 | 0.034 | 2.34 | 2.74 | no | no | no | n.a. |
| Miglustat | new signal | 7.14 | 16.74 | 19.59 | 2.34 | 2.74 | no | yes | yes | lysosomal storage disease therapy |
| Crizanlizumab | new signal | 5.51 | 12.91 | 15.11 | 2.34 | 2.74 | no | yes | yes | sickle-cell disease / thalassaemia |
| Belumosudil | new signal | 3.23 | 7.57 | 8.86 | 2.34 | 2.74 | no | yes | yes | transplant / autoimmune immunosuppressant |
| Taxotere | new signal | 3.23 | 7.56 | 8.85 | 2.34 | 2.74 | no | yes | yes | breast / prostate cancer endocrine or targeted therapy |
| Raltegravir | new signal | 3.09 | 7.23 | 8.47 | 2.34 | 2.74 | no | yes | yes | other |
| Betamethasone | new signal | 3.07 | 7.19 | n.a. | 2.34 | n.a. | no | yes | no | other |
| Raloxifene | new signal | 2.96 | 6.93 | 8.12 | 2.35 | 2.74 | no | yes | yes | other |
| Vincristine | new signal | 2.70 | 6.34 | 7.42 | 2.35 | 2.74 | no | yes | yes | leukaemia / lymphoma / myeloma chemotherapy |
| Tretinoin | new signal | 2.63 | 6.16 | 7.21 | 2.34 | 2.74 | no | yes | yes | leukaemia / lymphoma / myeloma chemotherapy |
| Hydroxyurea | new signal | 2.63 | 6.15 | 7.20 | 2.34 | 2.74 | no | yes | yes | sickle-cell disease / thalassaemia |
| Azathioprine | new signal | 2.52 | 5.92 | 6.92 | 2.34 | 2.74 | no | yes | yes | transplant / autoimmune immunosuppressant |
| Exemestane | new signal | 2.44 | 5.73 | 6.71 | 2.34 | 2.74 | no | yes | yes | breast / prostate cancer endocrine or targeted therapy |
| Lymphocyte Immune Globulin, Anti-Thymocyte Globulin | new signal | 2.31 | 5.41 | 6.33 | 2.34 | 2.74 | no | yes | yes | transplant / autoimmune immunosuppressant |
| Etoposide | new signal | 2.26 | 5.30 | 6.21 | 2.35 | 2.75 | no | yes | yes | leukaemia / lymphoma / myeloma chemotherapy |
| Anastrozole | new signal | 2.22 | 5.21 | 6.10 | 2.35 | 2.75 | no | yes | yes | breast / prostate cancer endocrine or targeted therapy |
| Cyclophosphamide | new signal | 2.20 | 5.18 | 6.07 | 2.35 | 2.75 | no | yes | yes | leukaemia / lymphoma / myeloma chemotherapy |
| Sirolimus | new signal | 2.15 | 5.04 | 5.90 | 2.35 | 2.74 | no | yes | yes | transplant / autoimmune immunosuppressant |
| Doxorubicin | new signal | 2.09 | 4.93 | 5.77 | 2.35 | 2.76 | no | yes | yes | leukaemia / lymphoma / myeloma chemotherapy |
| Mabthera | new signal | 2.04 | 4.78 | 5.60 | 2.34 | 2.74 | no | yes | yes | leukaemia / lymphoma / myeloma chemotherapy |
| Bevacizumab | new signal | 2.02 | 4.77 | 5.60 | 2.37 | 2.78 | no | yes | yes | breast / prostate cancer endocrine or targeted therapy |
| Melphalan | new signal | 1.94 | 4.55 | 5.33 | 2.34 | 2.74 | no | yes | yes | leukaemia / lymphoma / myeloma chemotherapy |
| Cyclosporine | new signal | 1.86 | 4.40 | 5.16 | 2.36 | 2.77 | no | yes | yes | transplant / autoimmune immunosuppressant |
| Letrozole | new signal | 1.77 | 4.16 | 4.87 | 2.35 | 2.75 | no | yes | yes | breast / prostate cancer endocrine or targeted therapy |
| Goserelin | new signal | 1.70 | 3.99 | 4.66 | 2.34 | 2.74 | no | yes | yes | breast / prostate cancer endocrine or targeted therapy |
| Fludarabine | new signal | 1.68 | 3.94 | 4.61 | 2.34 | 2.74 | no | yes | yes | leukaemia / lymphoma / myeloma chemotherapy |
| Isopropyl Alcohol | new signal | 1.67 | 3.92 | 4.59 | 2.34 | 2.74 | no | yes | yes | other (unmapped product name) |
| Thalidomide | new signal | 1.64 | 3.86 | 4.52 | 2.35 | 2.75 | no | yes | yes | leukaemia / lymphoma / myeloma chemotherapy |
| Interferon Beta-1A | new signal | 1.60 | 3.81 | 4.48 | 2.38 | 2.80 | no | yes | yes | rheumatology / neurology immunomodulator |
| Imatinib | new signal | 1.60 | 3.77 | 4.42 | 2.36 | 2.76 | no | yes | yes | breast / prostate cancer endocrine or targeted therapy |
| Romosozumab | new signal | 1.59 | 3.73 | 4.37 | 2.35 | 2.74 | no | yes | yes | osteoporosis anabolic (antiresorptive co-prescription) |
| Fulvestrant | new signal | 1.56 | 3.66 | 4.28 | 2.34 | 2.74 | no | yes | yes | breast / prostate cancer endocrine or targeted therapy |
| Duragesic-100 | new signal | 1.53 | 3.60 | 4.21 | 2.34 | 2.74 | no | yes | yes | other (opioid analgesic) |
| Blinatumomab | new signal | 1.51 | 3.53 | 4.13 | 2.34 | 2.74 | no | yes | yes | leukaemia / lymphoma / myeloma chemotherapy |
| Deferasirox | new signal | 1.46 | 3.42 | 4.01 | 2.35 | 2.75 | no | yes | yes | sickle-cell disease / thalassaemia |
| Cytarabine | new signal | 1.45 | 3.41 | 3.99 | 2.35 | 2.74 | no | yes | yes | leukaemia / lymphoma / myeloma chemotherapy |
| Ferric Carboxymaltose | new signal | 1.40 | 3.27 | 3.83 | 2.34 | 2.74 | no | no | yes | other (intravenous iron) |
| Tacrolimus | new signal | 1.36 | 3.20 | 3.76 | 2.36 | 2.76 | no | yes | yes | transplant / autoimmune immunosuppressant |
| Hydroxychloroquine | new signal | 1.33 | 3.12 | 3.65 | 2.35 | 2.75 | no | yes | yes | rheumatology / neurology immunomodulator |
| Alectinib | new signal | 1.33 | 3.11 | 3.64 | 2.34 | 2.74 | no | no | yes | breast / prostate cancer endocrine or targeted therapy |
| Leflunomide | new signal | 1.27 | 2.99 | 3.50 | 2.35 | 2.74 | no | yes | yes | rheumatology / neurology immunomodulator |
| Ritonavir | new signal | 1.25 | 2.96 | 3.46 | 2.36 | 2.76 | no | yes | yes | antiretroviral booster (HIV) |
| Upadacitinib | new signal | 1.20 | 2.83 | 3.32 | 2.36 | 2.76 | no | yes | yes | rheumatology / neurology immunomodulator |
| Methotrexate | new signal | 1.13 | 2.67 | 3.13 | 2.37 | 2.78 | no | yes | yes | leukaemia / lymphoma / myeloma chemotherapy |
| Somatropin | new signal | 1.07 | 2.52 | 2.96 | 2.36 | 2.76 | no | yes | yes | growth hormone |
| Rituximab | new signal | 1.05 | 2.47 | 2.89 | 2.36 | 2.77 | no | yes | yes | leukaemia / lymphoma / myeloma chemotherapy |
| Teriparatide | new signal | 0.92 | 2.18 | 2.55 | 2.36 | 2.76 | no | no | yes | osteoporosis anabolic (antiresorptive co-prescription) |
| Pamidronate | signal (filtered out) | 194.3 | n.a. | n.a. | n.a. | n.a. | yes | no | no | n.a. |
| Tenofovir Disoproxil | signal (filtered out) | 157.9 | n.a. | n.a. | n.a. | n.a. | yes | no | no | n.a. |
| Alendronate | signal (filtered out) | 69.46 | n.a. | n.a. | n.a. | n.a. | yes | no | no | n.a. |
| Zoledronic Acid | signal (filtered out) | 49.74 | n.a. | n.a. | n.a. | n.a. | yes | no | no | n.a. |
| Emtricitabine | signal (filtered out) | 45.76 | n.a. | n.a. | n.a. | n.a. | yes | no | no | n.a. |
| Efavirenz | signal (filtered out) | 38.90 | n.a. | n.a. | n.a. | n.a. | yes | no | no | n.a. |
| Elvitegravir | signal (filtered out) | 23.62 | n.a. | n.a. | n.a. | n.a. | yes | no | no | n.a. |
| Cobicistat | signal (filtered out) | 18.01 | n.a. | n.a. | n.a. | n.a. | yes | no | no | n.a. |
| Taliglucerase Alfa | signal (filtered out) | 17.84 | 41.82 | 48.93 | 2.34 | 2.74 | yes | yes | yes | n.a. |
| Nelfinavir | signal (filtered out) | 16.25 | 38.08 | 44.56 | 2.34 | 2.74 | yes | yes | yes | n.a. |
| Ibandronate | signal (filtered out) | 11.30 | n.a. | n.a. | n.a. | n.a. | yes | no | no | n.a. |
| Mitoxantrone | signal (filtered out) | 9.27 | 21.73 | 25.42 | 2.34 | 2.74 | yes | yes | yes | n.a. |
| Risedronate | signal (filtered out) | 8.38 | n.a. | n.a. | n.a. | n.a. | yes | no | no | n.a. |
| Rilpivirine | signal (filtered out) | 7.47 | n.a. | n.a. | n.a. | n.a. | yes | no | no | n.a. |
| Lopinavir | signal (filtered out) | 7.35 | 17.26 | 20.21 | 2.35 | 2.75 | yes | yes | yes | n.a. |
| Glutamine | signal (filtered out) | 5.73 | 13.44 | 15.73 | 2.34 | 2.74 | yes | yes | yes | n.a. |
| Zidovudine | signal (filtered out) | 4.69 | 11.01 | 12.89 | 2.35 | 2.75 | yes | yes | yes | n.a. |
| Mercaptopurine | signal (filtered out) | 3.98 | 9.34 | 10.93 | 2.34 | 2.74 | yes | yes | yes | n.a. |
| Nevirapine | signal (filtered out) | 3.92 | 9.20 | 10.77 | 2.35 | 2.75 | yes | yes | yes | n.a. |
| Denosumab | signal (filtered out) | 2.62 | n.a. | n.a. | n.a. | n.a. | yes | no | no | n.a. |
| Indinavir | survivor | 60.95 | 142.9 | 167.3 | 2.34 | 2.74 | yes | yes | yes | n.a. |
| Imiglucerase | survivor | 23.57 | 55.57 | 65.15 | 2.36 | 2.76 | yes | yes | yes | n.a. |
| Cholecalciferol | survivor | 22.98 | n.a. | n.a. | n.a. | n.a. | yes | no | no | n.a. |
| Stavudine | survivor | 19.92 | 46.70 | 54.65 | 2.34 | 2.74 | yes | yes | yes | n.a. |
| Velaglucerase Alfa | survivor | 16.07 | 37.71 | 44.14 | 2.35 | 2.75 | yes | yes | yes | n.a. |
| Asparaginase | survivor | 13.33 | 31.29 | 36.63 | 2.35 | 2.75 | yes | yes | yes | n.a. |
| Methylprednisolone | survivor | 12.42 | 29.75 | n.a. | 2.39 | n.a. | yes | yes | no | n.a. |
| Arsenic Trioxide | survivor | 9.35 | 21.92 | 25.65 | 2.34 | 2.74 | yes | yes | yes | n.a. |
| Prednisone | survivor | 8.11 | 19.43 | n.a. | 2.40 | n.a. | yes | yes | no | n.a. |
| Adefovir | survivor | 8.00 | 18.76 | 21.96 | 2.34 | 2.74 | yes | yes | yes | n.a. |
| Prednisolone | survivor | 7.99 | 19.09 | n.a. | 2.39 | n.a. | yes | yes | no | n.a. |
| Atazanavir | survivor | 6.94 | 16.27 | 19.05 | 2.35 | 2.75 | yes | yes | yes | n.a. |
| Satralizumab | survivor | 6.53 | 15.29 | 17.89 | 2.34 | 2.74 | yes | yes | yes | n.a. |
| Pegaspargase | survivor | 5.94 | 13.93 | 16.30 | 2.35 | 2.75 | yes | yes | yes | n.a. |
| Eliglustat | survivor | 5.68 | 13.31 | 15.57 | 2.34 | 2.74 | yes | yes | yes | n.a. |
| Dexamethasone | survivor | 5.52 | 13.16 | n.a. | 2.39 | n.a. | yes | yes | no | n.a. |
| Voxelotor | survivor | 5.06 | 11.94 | 14.01 | 2.36 | 2.77 | yes | yes | yes | n.a. |
| Hydrocortisone | survivor | 4.33 | 10.17 | n.a. | 2.35 | n.a. | yes | yes | no | n.a. |
| Interferon alfa-2b | survivor | 3.84 | 8.99 | 10.52 | 2.34 | 2.74 | yes | yes | yes | n.a. |
| Abacavir | survivor | 3.76 | 8.84 | 10.35 | 2.35 | 2.75 | yes | yes | yes | n.a. |
| Lamivudine | survivor | 3.47 | 8.23 | 9.65 | 2.37 | 2.78 | yes | yes | yes | n.a. |
| Tenofovir | survivor | 3.25 | 7.24 | 8.48 | 2.23 | 2.61 | yes | no | no | n.a. |
| Darunavir | survivor | 3.19 | 9.45 | 11.06 | 2.96 | 3.47 | yes | yes | yes | n.a. |
| Triamcinolone | survivor | 2.99 | 7.02 | n.a. | 2.35 | n.a. | yes | yes | no | n.a. |
| Testosterone | survivor | 2.75 | 6.50 | 7.62 | 2.36 | 2.77 | yes | yes | yes | n.a. |
| Dolutegravir | survivor | 2.68 | 6.47 | 7.58 | 2.41 | 2.82 | yes | yes | yes | n.a. |
| Mycophenolate Mofetil | survivor | 2.58 | 6.09 | 7.15 | 2.36 | 2.77 | yes | yes | yes | n.a. |

**Supplementary Table S6. Characteristics of the three databases and cross-database concordance**

Structural differences between FAERS, Canada Vigilance and JADER that bear on the interpretation of the external replication, followed by the concordance actually observed. Reporter-channel percentages are computed from each database (FAERS occupation code; Canada Vigilance reporter-type field); The JADER reporter-qualification field is tabulated from the demographics file rather than assumed, because a consumer and, rarely, a lawyer qualification do occur there. Concordance was assessed on direction and four-criterion status, not on equality of effect size, because the background composition of each database differs. JPI = jaw-propensity index; ROR = reporting odds ratio; PT = MedDRA preferred term.

| **Characteristic** | **FAERS** | **Canada Vigilance** | **JADER** |
| --- | --- | --- | --- |
| Regulator / custodian | U.S. Food and Drug Administration | Health Canada (MedEffect Canada) | Pharmaceuticals and Medical Devices Agency (PMDA), Japan |
| Period analysed | 2004Q1–2026Q2 (90 quarterly releases) | Database inception to 30 November 2024 (bulk extract) | June 2026 release; reports labelled to the end of Japanese fiscal-year 2025, that is to March 2026 |
| Reports analysed | 20,690,895 deduplicated reports | 1,154,017 reports | 1,037,161 cases |
| Non-jaw osteonecrosis cases (primary definition) | 24,087 | 496 | 1,362 |
| Outcome vocabulary | Five explicit non-jaw PTs + site-unspecified OSTEONECROSIS, report-level adjudication (Scheme C) | Corresponding English MedDRA vocabulary, the same explicit non-jaw terms and a database-specific oral co-event exclusion corresponding to rule R1; rule R2 needs an indication table and is FAERS-only, and rule R3 removes nothing beyond R1 | MedDRA/J equivalents of the same vocabulary, with 骨壊死 (site unspecified) adjudicated by the same oral co-event rule (R1), plus the explicit non-jaw terms 骨髄壊死, ペルテス病 and 骨梗塞; rules R2 and R3 are FAERS-only |
| Jaw term used for the JPI | OSTEONECROSIS OF JAW | OSTEONECROSIS OF JAW | 顎骨壊死 (osteonecrosis of jaw) |
| Drug role | Primary suspect (role_cod = PS), RxNorm ingredient mapping | Suspect role in report_drug, same ingredient map | 被疑薬 (suspect) only; Japanese generic names mapped by substring; systemic routes only for glucocorticoids |
| Reporter channels | Consumers 43.7%, physicians 22.0%, other/health professionals 18.6%, pharmacists 6.3%, lawyers 2.6% of all reports; lawyers 25.8% of osteonecrosis cases | Consumers / non-health professionals 21% of all reports and 16% of osteonecrosis cases; lawyer-type reports 0.3% of all reports | Forwarded mainly by marketing-authorisation holders and medical institutions; a consumer qualification (消費者等) appears on 8.4% of reports and a lawyer qualification (弁護士) on 0.02% (198 reports) |
| Litigation context for tenofovir disoproxil | U.S. mass tort (Gilead Tenofovir Cases) active during the study window | Class proceedings filed in British Columbia and Quebec in 2021 | No Japanese proceedings identified |
| Lawyer-filed tenofovir disoproxil reports | 5,937 of 7,027 osteonecrosis cases (84.5%) | 2 of 1,127 tenofovir reports | no lawyer-filed report identified |
| Underlying disease / indication recorded | INDI table (MedDRA PT per drug) | report_drug_indication table (not used) | 原疾患等 table (used for the within-HIV restriction) |
| Intermediate-set drugs testable (≥ 3 cases) / replicated (four criteria) | n.a. | 5 / 3 | 13 / 9 |
| Glucocorticoid class ROR (95% CI) | 3.0 to 12.4 per drug (Table 2) | 10.3 (8.3–12.8); 102 cases | 54.4 (48.3–61.3); 988 cases |
| Tenofovir disoproxil ROR (95% CI) | 157.9 (153.4–162.6) all reporters; 6.33 (5.38–7.45) health professionals; 27.5 (25.2–30.1) within HIV indication | 6.24 (2.00–19.4); 3 cases | 10.5 (6.60–16.8); 18 cases; within HIV indication 2.75 (1.26–6.00) |
| JPI concordance with FAERS (drugs with ≥ 3 cases) | n.a. | Pearson r = 0.985 (n = 11) | Pearson r = 0.960 (n = 24) |
| Antiresorptive JPI (alendronate / pamidronate / zoledronic acid) | 0.50 / 0.55 / 0.71 | 0.66 / 0.67 / 0.74 | 0.98 / 0.97 / 0.99 |

**Supplementary Table S7. Descriptive characteristics of the final case cohort (n = 24,087)**

The onset-year rows give the event (onset) year rather than the report year, since all 24,087 reports carry FDA report dates within 2004Q1–2026Q2, whereas the onset year is recorded for 15,146 (62.9%, including partial year-only dates) and legitimately includes pre-2004 onsets from retrospectively reported events. Sex percentages are among all reports (unknown 8.6%); among reports with a known sex, 47.7% are male.

**Onset year (event date)**

| **Category** | **n** | **%** |
| --- | --- | --- |
| 2018 | 1,018 | 4.2% |
| 2019 | 969 | 4.0% |
| 2017 | 962 | 4.0% |
| 2015 | 920 | 3.8% |
| 2016 | 906 | 3.8% |
| 2006 | 793 | 3.3% |
| 2014 | 783 | 3.3% |
| 2005 | 765 | 3.2% |
| 2008 | 752 | 3.1% |
| 2007 | 739 | 3.1% |
| 2009 | 734 | 3.0% |
| 2013 | 727 | 3.0% |
| 2012 | 681 | 2.8% |
| 2010 | 656 | 2.7% |
| 2004 | 595 | 2.5% |
| 2011 | 554 | 2.3% |
| 2020 | 450 | 1.9% |
| 2021 | 337 | 1.4% |
| 2003 | 293 | 1.2% |
| 2022 | 279 | 1.2% |
| 2023 | 251 | 1.0% |
| 2024 | 198 | 0.8% |
| 2002 | 180 | 0.7% |
| 2025 | 153 | 0.6% |
| 2001 | 108 | 0.4% |
| 2000 | 91 | 0.4% |
| 1999 | 69 | 0.3% |
| 1998 | 40 | 0.2% |
| 1997 | 31 | 0.1% |
| 1996 | 27 | 0.1% |
| 2026 | 24 | 0.1% |
| 1990 | 19 | 0.1% |
| 1995 | 10 | 0.0% |
| 1988 | 5 | 0.0% |
| 1993 | 5 | 0.0% |
| 1994 | 3 | 0.0% |
| 1991 | 3 | 0.0% |
| 1984 | 3 | 0.0% |
| 1987 | 3 | 0.0% |
| 1992 | 2 | 0.0% |
| 1973 | 2 | 0.0% |
| 1971 | 1 | 0.0% |
| 1980 | 1 | 0.0% |
| 1972 | 1 | 0.0% |
| 1983 | 1 | 0.0% |
| 1989 | 1 | 0.0% |
| 1985 | 1 | 0.0% |

**Age group**

| **Category** | **n** | **%** |
| --- | --- | --- |
| Unknown | 8,396 | 34.9% |
| 40–59 | 6,693 | 27.8% |
| 60–79 | 4,740 | 19.7% |
| 18–39 | 2,740 | 11.4% |
| <18 | 818 | 3.4% |
| ≥80 | 700 | 2.9% |

**Sex**

| **Category** | **n** | **%** |
| --- | --- | --- |
| F | 11,519 | 47.8% |
| M | 10,508 | 43.6% |
| Unknown | 2,060 | 8.6% |

**Reporter**

| **Category** | **n** | **%** |
| --- | --- | --- |
| Physician | 6,851 | 28.4% |
| Lawyer | 6,214 | 25.8% |
| Consumer | 3,764 | 15.6% |
| Other HCP | 3,319 | 13.8% |
| Unknown | 2,168 | 9.0% |
| Health professional | 1,266 | 5.3% |
| Pharmacist | 499 | 2.1% |
| Registered nurse | 6 | 0.0% |

**Systemic GC co-exposure**

| **Category** | **n** | **%** |
| --- | --- | --- |
| No | 19,400 | 80.5% |
| Yes | 4,687 | 19.5% |

**Reporting country (top 12)**

| **Country** | **n** | **%** |
| --- | --- | --- |
| UNITED STATES | 13,526 | 56.2% |
| FRANCE | 1,127 | 4.7% |
| GERMANY | 1,019 | 4.2% |
| UNKNOWN | 967 | 4.0% |
| JAPAN | 964 | 4.0% |
| ITALY | 832 | 3.5% |
| UNITED KINGDOM | 713 | 3.0% |
| CANADA | 573 | 2.4% |
| CHINA | 338 | 1.4% |
| AUSTRALIA | 336 | 1.4% |
| SPAIN | 330 | 1.4% |
| BRAZIL | 310 | 1.3% |

**Supplementary Table S8. Complete list of the 47 four-criterion signals (adjudicated scheme C, primary scan)**

All drugs meeting all four disproportionality criteria, with the 95% CI of the ROR, the jaw-propensity index (JPI), the lawyer-sourced share of the drug's cases, the health-professional-only (HCP) ROR, and the two pre-specified pass flags (pass jaw = JPI < 0.50; pass reporter = HCP-only signal retained under all four criteria with HCP/all ROR ratio at least 0.5). The survives column marks the 27 signals of the intermediate set (Table 2).

| **Ingredient** | **a** | **ROR** | **95% CI** | **JPI** | **Lawyer-sourced** | **a (HCP)** | **HCP ROR** | **Ratio HCP/all** | **Pass jaw** | **Pass reporter** | **Survives** |
| --- | --- | --- | --- | --- | --- | --- | --- | --- | --- | --- | --- |
| Tenofovir Disoproxil | 7,027 | 157.90 | 153.38–162.55 | 0.001 | 84% | 148 | 6.33 | 0.04 | yes | no | no |
| Zoledronic Acid | 2,945 | 49.74 | 47.81–51.73 | 0.713 | 2% | 2,379 | 75.99 | 1.53 | no | yes | no |
| Emtricitabine | 2,579 | 45.76 | 43.89–47.70 | 0.004 | 79% | 146 | 4.79 | 0.10 | yes | no | no |
| Alendronate | 2,457 | 69.46 | 66.53–72.51 | 0.502 | 1% | 1,404 | 75.11 | 1.08 | no | yes | no |
| Pamidronate | 684 | 194.25 | 178.65–211.23 | 0.554 | 11% | 466 | 202.2 | 1.04 | no | yes | no |
| Efavirenz | 588 | 38.90 | 35.78–42.29 | 0.007 | 80% | 16 | 2.22 | 0.06 | yes | no | no |
| Denosumab | 481 | 2.62 | 2.40–2.87 | 0.943 | 0% | 366 | 2.88 | 1.10 | no | yes | no |
| Prednisone | 392 | 8.11 | 7.33–8.96 | 0.157 | 0% | 272 | 6.81 | 0.84 | yes | yes | yes |
| Methylprednisolone | 377 | 12.42 | 11.21–13.76 | 0.058 | 2% | 246 | 9.81 | 0.79 | yes | yes | yes |
| Prednisolone | 336 | 7.99 | 7.17–8.90 | 0.276 | 4% | 289 | 8.32 | 1.04 | yes | yes | yes |
| Dexamethasone | 312 | 5.52 | 4.93–6.17 | 0.235 | 0% | 265 | 5.42 | 0.98 | yes | yes | yes |
| Ibandronate | 266 | 11.30 | 10.00–12.76 | 0.649 | 2% | 205 | 24.73 | 2.19 | no | yes | no |
| Mycophenolate Mofetil | 159 | 2.58 | 2.21–3.01 | 0.117 | 0% | 129 | 2.44 | 0.95 | yes | yes | yes |
| Cobicistat | 155 | 18.01 | 15.35–21.13 | 0.000 | 72% | 15 | 2.63 | 0.15 | yes | no | no |
| Elvitegravir | 152 | 23.62 | 20.09–27.76 | 0.000 | 73% | 12 | 3.05 | 0.13 | yes | no | no |
| Rilpivirine | 137 | 7.47 | 6.31–8.84 | 0.000 | 74% | 13 | 0.99 | 0.13 | yes | no | no |
| Voxelotor | 128 | 5.06 | 4.25–6.03 | 0.000 | 1% | 81 | 23.21 | 4.58 | yes | yes | yes |
| Testosterone | 126 | 2.75 | 2.31–3.28 | 0.038 | 2% | 97 | 8.94 | 3.25 | yes | yes | yes |
| Imiglucerase | 118 | 23.57 | 19.62–28.31 | 0.017 | 0% | 97 | 28.16 | 1.19 | yes | yes | yes |
| Lamivudine | 102 | 3.47 | 2.86–4.22 | 0.019 | 0% | 59 | 3.01 | 0.87 | yes | yes | yes |
| Cholecalciferol | 71 | 22.98 | 18.14–29.09 | 0.466 | 1% | 56 | 24.22 | 1.05 | yes | yes | yes |
| Risedronate | 60 | 8.38 | 6.50–10.81 | 0.779 | 5% | 46 | 11.20 | 1.34 | no | yes | no |
| Triamcinolone | 59 | 2.99 | 2.31–3.86 | 0.000 | 0% | 48 | 4.71 | 1.58 | yes | yes | yes |
| Lopinavir | 52 | 7.35 | 5.59–9.66 | 0.000 | 44% | 15 | 2.81 | 0.38 | yes | no | no |
| Hydrocortisone | 51 | 4.33 | 3.29–5.70 | 0.038 | 0% | 38 | 5.06 | 1.17 | yes | yes | yes |
| Abacavir | 46 | 3.76 | 2.82–5.03 | 0.042 | 0% | 31 | 4.44 | 1.18 | yes | yes | yes |
| Dolutegravir | 45 | 2.68 | 2.00–3.60 | 0.000 | 0% | 36 | 3.88 | 1.45 | yes | yes | yes |
| Asparaginase | 43 | 13.33 | 9.86–18.02 | 0.000 | 0% | 42 | 15.98 | 1.20 | yes | yes | yes |
| Velaglucerase Alfa | 36 | 16.07 | 11.55–22.35 | 0.000 | 0% | 21 | 16.93 | 1.05 | yes | yes | yes |
| Zidovudine | 35 | 4.69 | 3.37–6.54 | 0.000 | 0% | 17 | 3.22 | 0.69 | yes | no | no |
| Atazanavir | 33 | 6.94 | 4.92–9.77 | 0.000 | 0% | 22 | 6.47 | 0.93 | yes | yes | yes |
| Pegaspargase | 30 | 5.94 | 4.15–8.50 | 0.000 | 3% | 26 | 5.15 | 0.87 | yes | yes | yes |
| Nevirapine | 29 | 3.92 | 2.72–5.65 | 0.000 | 0% | 16 | 3.32 | 0.85 | yes | no | no |
| Glutamine | 22 | 5.73 | 3.77–8.72 | 0.000 | 0% | 0 | n.a. | n.a. | yes | no | no |
| Indinavir | 22 | 60.95 | 39.54–93.94 | 0.000 | 0% | 16 | 54.45 | 0.89 | yes | yes | yes |
| Darunavir | 21 | 3.19 | 2.08–4.90 | 0.000 | 0% | 19 | 3.40 | 1.06 | yes | yes | yes |
| Tenofovir | 19 | 3.25 | 2.07–5.10 | 0.174 | 0% | 17 | 3.61 | 1.11 | yes | yes | yes |
| Stavudine | 19 | 19.92 | 12.64–31.40 | 0.000 | 0% | 17 | 21.31 | 1.07 | yes | yes | yes |
| Adefovir | 16 | 8.00 | 4.89–13.10 | 0.000 | 0% | 13 | 8.56 | 1.07 | yes | yes | yes |
| Interferon alfa-2b | 14 | 3.84 | 2.27–6.49 | 0.000 | 0% | 13 | 4.81 | 1.25 | yes | yes | yes |
| Arsenic Trioxide | 14 | 9.35 | 5.52–15.84 | 0.417 | 0% | 13 | 8.79 | 0.94 | yes | yes | yes |
| Mercaptopurine | 12 | 3.98 | 2.26–7.03 | 0.000 | 0% | 8 | 3.65 | 0.92 | yes | no | no |
| Nelfinavir | 11 | 16.25 | 8.95–29.51 | 0.000 | 9% | 5 | 9.30 | 0.57 | yes | no | no |
| Satralizumab | 11 | 6.53 | 3.60–11.81 | 0.000 | 0% | 10 | 8.73 | 1.34 | yes | yes | yes |
| Taliglucerase Alfa | 10 | 17.84 | 9.54–33.38 | 0.000 | 0% | 6 | 16.99 | 0.95 | yes | no | no |
| Eliglustat | 10 | 5.68 | 3.05–10.58 | 0.000 | 0% | 9 | 6.85 | 1.21 | yes | yes | yes |
| Mitoxantrone | 9 | 9.27 | 4.81–17.89 | 0.182 | 0% | 6 | 7.28 | 0.78 | yes | no | no |

**Supplementary Table S9. Vocabulary audit of the antiresorptive stratum of the conservative scheme (A)**

Among reports carrying an explicit non-jaw preferred term (Scheme A) with an antiresorptive as suspect drug, the table gives the share carried by ASEPTIC NECROSIS BONE, the share co-reporting an explicit jaw-site term or any oral/dental term, the share with an oncology indication (multiple myeloma, metastatic disease, breast or prostate cancer, malignant neoplasm, carcinoma) and the most frequent indications. Rules R1–R3 of the adjudicated scheme never operate on these reports; the audit shows why the explicit vocabulary nonetheless cannot be read as verified extra-oral disease.

| **Ingredient** | **Scheme A cases** | **ASEPTIC NECROSIS BONE (n)** | **Share** | **Explicit jaw PT co-reported** | **Any oral/dental term co-reported** | **Oncology indication** | **Most frequent indications (n)** |
| --- | --- | --- | --- | --- | --- | --- | --- |
| Pamidronate | 146 | 141 | 97% | 3.4% | 72.6% | 71% | MULTIPLE MYELOMA (50); METASTASES TO BONE (23); BREAST CANCER (18); BREAST CANCER METASTATIC (11); OSTEOPOROSIS (5); PLASMACYTOMA (5) |
| Zoledronic Acid | 415 | 401 | 97% | 0.7% | 56.1% | 76% | METASTASES TO BONE (128); MULTIPLE MYELOMA (112); BREAST CANCER METASTATIC (37); BREAST CANCER (22); PAIN (17); PROSTATE CANCER (13) |
| Alendronate | 32 | 9 | 28% | 12.5% | 43.8% | 6% | OSTEOPOROSIS (17); OSTEOPENIA (10); OSTEOPOROSIS PROPHYLAXIS (5); HYPERTENSION (3); PRODUCT USED FOR UNKNOWN INDICATION (3); GASTROOESOPHAGEAL REFLUX DISEASE (2) |
| Ibandronate | 1 | 0 | 0% | 0.0% | 0.0% | 0% | n.a. |
| Risedronate | 0 | 0 | 0% | 0.0% | 0.0% | 0% | n.a. |
| Denosumab | 14 | 0 | 0% | 0.0% | 0.0% | 14% | PRODUCT USED FOR UNKNOWN INDICATION (6); OSTEOPOROSIS (5); METASTASES TO BONE (2); OSTEOPOROSIS POSTMENOPAUSAL (1); BREAST CANCER STAGE IV (1); CROHN'S DISEASE (1) |

**Supplementary Table S10. Within-HIV-indication reporting odds ratios in JADER**

JADER records the underlying disease of each case (原疾患等). Reports whose underlying disease names HIV infection or acquired immunodeficiency syndrome were selected, and the ROR of each antiretroviral for 骨壊死 was recomputed inside that stratum, holding the HIV baseline constant, exactly as the FAERS indication-restricted analysis does for HIV-indication reports. The first row gives the ROR of the HIV indication itself over the whole JADER corpus.

| **Drug (within HIV stratum)** | **n reports** | **Osteonecrosis cases** | **ROR** | **95% CI** | **Four criteria** |
| --- | --- | --- | --- | --- | --- |
| HIV Infection (Indication) | 3,598 | 26 | 5.62 | 3.81–8.30 | yes |
| Tenofovir Disoproxil | 764 | 11 | 2.75 | 1.26–6.00 | no |
| Emtricitabine | 1,042 | 16 | 3.97 | 1.80–8.78 | no |
| Efavirenz | 407 | 1 | 0.31 | 0.04–2.31 | no |
| Lamivudine | 1,257 | 10 | 1.17 | 0.53–2.58 | no |
| Abacavir | 780 | 8 | 1.61 | 0.70–3.72 | no |
| Darunavir | 338 | 9 | 5.22 | 2.31–11.80 | no |
| Dolutegravir | 763 | 4 | 0.67 | 0.23–1.96 | no |
| Atazanavir | 220 | 0 | n.a. | n.a. | no |
| Raltegravir | 353 | 1 | 0.37 | 0.05–2.71 | no |
| Lopinavir | 220 | 3 | 2.02 | 0.60–6.77 | no |

**Supplementary Table S11. External validation in the Japanese JADER database**

Re-analysis of the 47 FAERS signals (plus the glucocorticoid class) in JADER, using the MedDRA/J counterparts of the FAERS outcome vocabulary, namely 骨壊死 (osteonecrosis, site unspecified) adjudicated by a Japanese oral co-event list, the explicit non-jaw terms 骨髄壊死, ペルテス病 and 骨梗塞, and 顎骨壊死 (osteonecrosis of jaw) as the jaw term, suspect-role exposure only, and systemic non-local routes for glucocorticoids. The FAERS-survivor column marks the 27 signals of the intermediate set; the JADER-significant column marks replication under all four disproportionality criteria. Of the 27, 13 had ≥ 3 Japanese cases and 9 replicated; the pooled glucocorticoid class replicated with ROR 54.4 (95% CI 48.3–61.3). The 95% CI upper bound is derived from the ROR and its lower bound on the log scale.

| **Ingredient** | **FAERS a** | **FAERS ROR** | **FAERS survivor** | **JADER n (drug)** | **JADER a** | **JADER ROR** | **JADER 95% CI** | **JADER EBGM05** | **JADER IC025** | **JADER significant** | **JADER JPI** |
| --- | --- | --- | --- | --- | --- | --- | --- | --- | --- | --- | --- |
| Tenofovir Disoproxil | 7,027 | 157.9 | no | 1,332 | 18 | 10.54 | 6.60–16.84 | 5.35 | 1.04 | yes | 0.00 |
| Zoledronic Acid | 2,945 | 49.74 | no | 3,977 | 16 | 3.10 | 1.89–5.07 | 1.82 | 0.16 | no | 0.99 |
| Emtricitabine | 2,579 | 45.76 | no | 1,297 | 21 | 12.70 | 8.22–19.61 | 6.57 | 1.29 | yes | 0.00 |
| Alendronate | 2,457 | 69.46 | no | 3,951 | 22 | 4.31 | 2.83–6.58 | 2.69 | 0.66 | yes | 0.98 |
| Pamidronate | 684 | 194.3 | no | 468 | 8 | 13.30 | 6.60–26.81 | 3.89 | 0.08 | yes | 0.97 |
| Efavirenz | 588 | 38.90 | no | 507 | 3 | 4.53 | 1.46–14.12 | 0.93 | -1.44 | no | 0.00 |
| Denosumab | 481 | 2.62 | no | 4,590 | 8 | 1.33 | 0.66–2.67 | 0.66 | -1.09 | no | 1.00 |
| Prednisone | 392 | 8.11 | yes | 39 | 0 | n.a. | n.a. | n.a. | n.a. | no | 0.00 |
| Methylprednisolone | 377 | 12.42 | yes | 6,635 | 207 | 28.70 | 24.70–33.34 | 20.01 | 3.54 | yes | 0.04 |
| Prednisolone | 336 | 7.99 | yes | 30,212 | 729 | 39.31 | 35.32–43.75 | 17.07 | 3.72 | yes | 0.23 |
| Dexamethasone | 312 | 5.52 | yes | 12,456 | 184 | 13.03 | 11.14–15.23 | 9.65 | 2.73 | yes | 0.23 |
| Ibandronate | 266 | 11.30 | no | 994 | 6 | 4.63 | 2.07–10.36 | 1.63 | -0.53 | no | 0.98 |
| Mycophenolate Mofetil | 159 | 2.58 | yes | 7,743 | 18 | 1.78 | 1.12–2.84 | 1.13 | -0.29 | no | 0.10 |
| Cobicistat | 155 | 18.01 | no | 145 | 1 | 5.28 | 0.74–37.80 | 0.25 | -2.99 | no | 0.00 |
| Elvitegravir | 152 | 23.62 | no | 93 | 1 | 8.27 | 1.15–59.39 | 0.28 | -2.99 | no | 0.00 |
| Rilpivirine | 137 | 7.47 | no | 225 | 0 | n.a. | n.a. | n.a. | n.a. | no | 0.00 |
| Voxelotor | 128 | 5.06 | yes | 0 | 0 | n.a. | n.a. | n.a. | n.a. | no | 0.00 |
| Testosterone | 126 | 2.75 | yes | 49 | 0 | n.a. | n.a. | n.a. | n.a. | no | 0.00 |
| Imiglucerase | 118 | 23.57 | yes | 30 | 0 | n.a. | n.a. | n.a. | n.a. | no | 0.00 |
| Lamivudine | 102 | 3.47 | yes | 2,020 | 15 | 5.74 | 3.45–9.57 | 3.06 | 0.55 | yes | 0.00 |
| Cholecalciferol | 71 | 22.98 | yes | 237 | 0 | n.a. | n.a. | n.a. | n.a. | no | 1.00 |
| Risedronate | 60 | 8.38 | no | 2,179 | 6 | 2.10 | 0.94–4.70 | 0.88 | -0.95 | no | 0.99 |
| Triamcinolone | 59 | 2.99 | yes | 432 | 12 | 21.91 | 12.31–38.99 | 6.85 | 0.70 | yes | 0.00 |
| Lopinavir | 52 | 7.35 | no | 512 | 6 | 9.05 | 4.04–20.28 | 2.51 | -0.37 | no | 0.00 |
| Hydrocortisone | 51 | 4.33 | yes | 1,336 | 46 | 28.03 | 20.79–37.80 | 15.92 | 2.44 | yes | 0.02 |
| Abacavir | 46 | 3.76 | yes | 963 | 9 | 7.22 | 3.73–13.94 | 2.87 | 0.11 | yes | 0.00 |
| Dolutegravir | 45 | 2.68 | yes | 913 | 4 | 3.35 | 1.25–8.97 | 0.98 | -1.14 | no | 0.00 |
| Asparaginase | 43 | 13.33 | yes | 1,221 | 103 | 75.71 | 61.42–93.34 | 41.53 | 3.65 | yes | 0.00 |
| Velaglucerase Alfa | 36 | 16.07 | yes | 30 | 0 | n.a. | n.a. | n.a. | n.a. | no | 0.00 |
| Zidovudine | 35 | 4.69 | no | 463 | 0 | n.a. | n.a. | n.a. | n.a. | no | 0.00 |
| Atazanavir | 33 | 6.94 | yes | 354 | 1 | 2.16 | 0.30–15.35 | 0.18 | -3.05 | no | 0.00 |
| Pegaspargase | 30 | 5.94 | yes | 54 | 0 | n.a. | n.a. | n.a. | n.a. | no | 0.00 |
| Nevirapine | 29 | 3.92 | no | 62 | 0 | n.a. | n.a. | n.a. | n.a. | no | 0.00 |
| Glutamine | 22 | 5.73 | no | 170 | 0 | n.a. | n.a. | n.a. | n.a. | no | 0.00 |
| Indinavir | 22 | 60.95 | yes | 56 | 0 | n.a. | n.a. | n.a. | n.a. | no | 0.00 |
| Darunavir | 21 | 3.19 | yes | 412 | 12 | 23.01 | 12.93–40.96 | 7.02 | 0.70 | yes | 0.00 |
| Tenofovir | 19 | 3.25 | yes | 0 | 0 | n.a. | n.a. | n.a. | n.a. | no | 0.00 |
| Stavudine | 19 | 19.92 | yes | 0 | 0 | n.a. | n.a. | n.a. | n.a. | no | 0.00 |
| Adefovir | 16 | 8.00 | yes | 530 | 7 | 10.23 | 4.84–21.60 | 3.04 | -0.14 | no | 0.00 |
| Interferon alfa-2b | 14 | 3.84 | yes | 0 | 0 | n.a. | n.a. | n.a. | n.a. | no | 0.00 |
| Arsenic Trioxide | 14 | 9.35 | yes | 496 | 0 | n.a. | n.a. | n.a. | n.a. | no | 0.00 |
| Mercaptopurine | 12 | 3.98 | no | 507 | 2 | 3.01 | 0.75–12.10 | 0.49 | -2.07 | no | 0.00 |
| Nelfinavir | 11 | 16.25 | no | 156 | 2 | 9.89 | 2.45–39.94 | 0.81 | -1.99 | no | 0.00 |
| Satralizumab | 11 | 6.53 | yes | 333 | 3 | 6.93 | 2.22–21.61 | 1.16 | -1.39 | no | 0.00 |
| Taliglucerase Alfa | 10 | 17.84 | no | 0 | 0 | n.a. | n.a. | n.a. | n.a. | no | 0.00 |
| Eliglustat | 10 | 5.68 | yes | 8 | 0 | n.a. | n.a. | n.a. | n.a. | no | 0.00 |
| Mitoxantrone | 9 | 9.27 | no | 591 | 3 | 3.89 | 1.25–12.10 | 0.85 | -1.47 | no | 0.00 |
| Glucocorticoids (class) | n.a. | n.a. | yes | 48,966 | 988 | 54.39 | 48.27–61.28 | 14.46 | 3.57 | yes | 0.22 |

**Supplementary Table S12. Indication-restricted analysis for every non-glucocorticoid drug of the intermediate set, together with the drugs analysed for continuity that are no longer in it, which are marked**

S12a. Confounding potential of each home indication, given as the ROR of the indication itself for osteonecrosis over the whole universe, with the MedDRA preferred-term substrings used to identify it in the INDI table.

| **Home indication** | **PT substrings** | **Reports with indication** | **Osteonecrosis cases** | **Indication ROR** | **95% CI** |
| --- | --- | --- | --- | --- | --- |
| transplant / immunosuppression | TRANSPLANT \| GRAFT \| IMMUNOSUPPRESS | 205,009 | 498 | 2.1 | 1.9–2.3 |
| acute lymphoblastic leukaemia | LYMPHOBLASTIC \| LYMPHOCYTIC LEUK | 128,882 | 404 | 2.7 | 2.5–3.0 |
| HIV infection | HIV \| IMMUNODEFICIENCY VIRUS \| ACQUIRED IMMUNODEFICIENCY \| ACQUIRED IMMUNE DEFICIENCY | 139,032 | 7,350 | 68.5 | 66.6–70.4 |
| Gaucher disease | GAUCHER | 7,761 | 174 | 19.8 | 17.0–23.0 |
| sickle-cell disease | SICKLE CELL \| SICKLING \| HAEMOGLOBIN S \| HEMOGLOBIN S | 24,750 | 173 | 6.1 | 5.2–7.1 |
| hypogonadism / androgen deficiency | HYPOGONAD \| TESTOSTERONE DEFICIENCY \| ANDROGEN DEFICIENCY | 5,185 | 50 | 8.4 | 6.3–11.1 |
| hepatitis B | HEPATITIS B | 17,450 | 120 | 6.0 | 5.0–7.1 |
| chronic viral hepatitis (B or C) | HEPATITIS B \| HEPATITIS C | 163,223 | 168 | 0.9 | 0.8–1.0 |
| neuromyelitis optica spectrum disorder | NEUROMYELITIS OPTICA | 6,612 | 35 | 4.6 | 3.3–6.4 |
| acute promyelocytic leukaemia | PROMYELOCYTIC | 2,941 | 25 | 7.4 | 5.0–10.9 |
| osteoporosis / vitamin D deficiency | OSTEOPOROSIS \| OSTEOPENIA \| VITAMIN D DEFICIENCY | 284,650 | 1,992 | 6.5 | 6.2–6.8 |

S12b. ROR of each drug recomputed inside reports carrying its home indication (disease baseline held constant). Cases with indication is the share of the drug's osteonecrosis cases that carry the indication. A within-indication ROR whose lower 95% bound is at or below 1 marks an indication signal, that is, no residual excess over the disease baseline was detected; a lower bound above 1 marks a residual within-indication signal, that is a candidate drug effect beyond the disease baseline; the significant flag marks a within-indication ROR with lower 95% bound > 1 and a ≥ 3. n.e. = not evaluable (zero cell).

| **Drug** | **Home indication** | **n (drug)** | **n (drug + indication)** | **a (within)** | **Cases with indication** | **Within-indication ROR** | **95% CI** | **Significant** |
| --- | --- | --- | --- | --- | --- | --- | --- | --- |
| Mycophenolate Mofetil | transplant / immunosuppression | 53,284 | 28,676 | 63 | 40% | 0.89 | 0.68–1.16 | no |
| Asparaginase | acute lymphoblastic leukaemia | 2,816 | 1,879 | 38 | 88% | 7.14 | 5.10–10.01 | yes |
| Pegaspargase | acute lymphoblastic leukaemia | 4,370 | 3,119 | 19 | 63% | 2.00 | 1.26–3.17 | yes |
| Tenofovir Disoproxil | HIV infection | 60,798 | 49,416 | 6,831 | 97% | 27.54 | 25.17–30.13 | yes |
| Tenofovir | HIV infection | 5,041 | 3,515 | 18 | 95% | 0.09 | 0.06–0.14 | no |
| Lamivudine | HIV infection | 25,386 | 16,107 | 72 | 71% | 0.07 | 0.06–0.09 | no |
| Abacavir | HIV infection | 10,547 | 7,297 | 41 | 89% | 0.10 | 0.07–0.13 | no |
| Dolutegravir | HIV infection | 14,444 | 9,087 | 31 | 69% | 0.06 | 0.04–0.08 | no |
| Zidovudine | HIV infection | 6,441 | 4,629 | 35 | 100% | 0.13 | 0.09–0.18 | no |
| Atazanavir | HIV infection | 4,120 | 2,491 | 25 | 76% | 0.18 | 0.12–0.27 | no |
| Nevirapine | HIV infection | 6,376 | 4,989 | 27 | 93% | 0.09 | 0.06–0.14 | no |
| Indinavir | HIV infection | 332 | 258 | 18 | 82% | 1.34 | 0.83–2.17 | no |
| Darunavir | HIV infection | 5,674 | 3,308 | 15 | 71% | 0.08 | 0.05–0.13 | no |
| Stavudine | HIV infection | 838 | 513 | 12 | 63% | 0.43 | 0.24–0.76 | no |
| Imiglucerase | Gaucher disease | 4,434 | 3,901 | 110 | 93% | 1.72 | 1.26–2.35 | yes |
| Velaglucerase Alfa | Gaucher disease | 1,961 | 1,714 | 34 | 94% | 0.85 | 0.58–1.25 | no |
| Taliglucerase Alfa | Gaucher disease | 491 | 371 | 8 | 80% | 0.96 | 0.47–1.96 | no |
| Eliglustat | Gaucher disease | 1,521 | 1,275 | 9 | 90% | 0.27 | 0.14–0.53 | no |
| Voxelotor | sickle-cell disease | 21,914 | 15,910 | 110 | 86% | 0.97 | 0.71–1.32 | no |
| Testosterone | hypogonadism / androgen deficiency | 39,510 | 3,200 | 44 | 35% | 4.60 | 1.96–10.81 | yes |
| Adefovir | hepatitis B | 1,732 | 1,333 | 12 | 75% | 1.35 | 0.74–2.45 | no |
| Interferon alfa-2b | chronic viral hepatitis (B or C) | 3,146 | 360 | 0 | 0% | n.e. | n.e. | no |
| Satralizumab | neuromyelitis optica spectrum disorder | 1,458 | 1,298 | 11 | 100% | 1.88 | 0.92–3.86 | no |
| Arsenic Trioxide | acute promyelocytic leukaemia | 1,299 | 751 | 12 | 86% | 2.72 | 1.24–5.99 | yes |
| Cholecalciferol | osteoporosis / vitamin D deficiency | 2,730 | 984 | 51 | 72% | 7.93 | 5.96–10.55 | yes |

S12c. Within-HIV-indication ROR of the antiretrovirals analysed in Table S12b, in which a drug no longer in the intermediate set is marked, after removing from the HIV stratum every report that names tenofovir disoproxil, emtricitabine or efavirenz as a suspect drug (the litigation cluster), leaving 75,386 HIV-indication reports with 477 osteonecrosis cases as the comparator.

| **Drug** | **n (drug, HIV stratum)** | **a** | **Within-HIV ROR** | **95% CI** | **Significant** |
| --- | --- | --- | --- | --- | --- |
| Tenofovir | 1,093 | 7 | 1.01 | 0.48–2.14 | no |
| Lamivudine | 15,603 | 70 | 0.66 | 0.51–0.85 | no |
| Abacavir | 7,297 | 41 | 0.88 | 0.64–1.21 | no |
| Dolutegravir | 8,833 | 29 | 0.49 | 0.33–0.71 | no |
| Zidovudine (not in intermediate set) | 4,629 | 35 | 1.21 | 0.86–1.71 | no |
| Atazanavir | 2,491 | 25 | 1.62 | 1.08–2.44 | yes |
| Nevirapine (not in intermediate set) | 4,989 | 27 | 0.85 | 0.57–1.25 | no |
| Indinavir | 258 | 18 | 12.20 | 7.49–19.87 | yes |
| Darunavir | 2,899 | 15 | 0.81 | 0.48–1.36 | no |
| Stavudine | 513 | 12 | 3.83 | 2.15–6.84 | yes |

**Supplementary Table S13. Complete Weibull time-to-onset results**

Per-drug Weibull shape parameter (α) with bootstrap 95% CI for the 47 four-criterion signals, which include the antiresorptive contrast drugs, with the classification of decreasing (α < 1), constant (α ≈ 1) and increasing (α > 1) hazard. The fitted median is the median of the Weibull distribution and the observed median that of the records themselves; where the two diverge, or where the onsets take few distinct values, the shape parameter is weakly identified. Onset records of the same drug that match on therapy start date, event date, sex and country are treated as reports of one case and counted once, because a case reported through several channels reaches FAERS as several case numbers. Drugs left with fewer than five such records are marked insufficient. Each onset is fitted as the one-day interval its day resolution implies, so a report recorded on the day of therapy start contributes the interval from 0 to 1 day rather than an exact zero, at which the Weibull density is unbounded for α < 1. Bootstrap resamples whose onset times all fell in the same positive one-day interval were recorded at infinity. All-zero resamples had a flat profile in α and were counted without assigning a shape value. The upper confidence limit was reported as not identified, and no hazard class was assigned, only when the 97.5th percentile fell among infinite values.

| **Ingredient** | **n onset records** | **n distinct values** | **Fitted Weibull median (days)** | **Observed median (days)** | **α** | **α 95% CI** | **Classification** |
| --- | --- | --- | --- | --- | --- | --- | --- |
| Tenofovir Disoproxil | 1,873 | 1,285 | 1,292 | 1,461 | 1.33 | 1.26–1.40 | delayed (alpha>1) |
| Zoledronic Acid | 1,279 | 730 | 524.0 | 586.0 | 1.17 | 1.11–1.24 | delayed (alpha>1) |
| Emtricitabine | 1,741 | 1,215 | 1,244 | 1,442 | 1.30 | 1.23–1.37 | delayed (alpha>1) |
| Alendronate | 599 | 431 | 808.0 | 1,090 | 0.93 | 0.84–1.02 | indeterminate |
| Pamidronate | 283 | 242 | 980.0 | 1,095 | 1.32 | 1.17–1.51 | delayed (alpha>1) |
| Efavirenz | 522 | 451 | 1,438 | 1,675 | 1.45 | 1.30–1.62 | delayed (alpha>1) |
| Denosumab | 101 | 92 | 302.9 | 397.0 | 0.80 | 0.65–1.02 | indeterminate |
| Prednisone | 119 | 104 | 264.6 | 335.0 | 0.74 | 0.60–0.93 | early (alpha<1) |
| Methylprednisolone | 107 | 88 | 173.4 | 304.0 | 0.54 | 0.44–0.67 | early (alpha<1) |
| Prednisolone | 91 | 76 | 167.1 | 182.0 | 0.56 | 0.47–0.67 | early (alpha<1) |
| Dexamethasone | 68 | 58 | 181.6 | 198.5 | 0.63 | 0.50–0.82 | early (alpha<1) |
| Ibandronate | 109 | 102 | 352.8 | 384.0 | 0.89 | 0.75–1.07 | indeterminate |
| Mycophenolate Mofetil | 22 | 22 | 211.2 | 265.0 | 0.83 | 0.58–1.39 | indeterminate |
| Cobicistat | 218 | 193 | 981.8 | 1,106 | 1.37 | 1.18–1.61 | delayed (alpha>1) |
| Elvitegravir | 218 | 193 | 981.8 | 1,106 | 1.37 | 1.18–1.61 | delayed (alpha>1) |
| Rilpivirine | 150 | 144 | 926.8 | 1,108 | 1.20 | 1.02–1.43 | delayed (alpha>1) |
| Voxelotor | 14 | 14 | 257.4 | 283.5 | 1.43 | 1.02–2.50 | delayed (alpha>1) |
| Testosterone | 4 | n.a. | n.a. | n.a. | n.a. | n.a. | insufficient |
| Imiglucerase | 37 | 37 | 1,168 | 1,948 | 0.95 | 0.61–1.79 | indeterminate |
| Lamivudine | 54 | 49 | 621.5 | 732.5 | 0.99 | 0.83–1.28 | indeterminate |
| Cholecalciferol | 45 | 40 | 351.5 | 394.0 | 0.62 | 0.45–0.88 | early (alpha<1) |
| Risedronate | 38 | 38 | 737.2 | 841.0 | 1.14 | 0.77–1.76 | indeterminate |
| Triamcinolone | 2 | n.a. | n.a. | n.a. | n.a. | n.a. | insufficient |
| Lopinavir | 38 | 35 | 702.7 | 706.0 | 0.94 | 0.67–1.31 | indeterminate |
| Hydrocortisone | 11 | 10 | 71 | 192.0 | 0.44 | 0.29–0.92 | early (alpha<1) |
| Abacavir | 23 | 20 | 298.2 | 239.0 | 1.11 | 0.87–1.58 | indeterminate |
| Dolutegravir | 12 | 11 | 223.7 | 197.5 | 0.88 | 0.68–1.48 | indeterminate |
| Asparaginase | 3 | n.a. | n.a. | n.a. | n.a. | n.a. | insufficient |
| Velaglucerase Alfa | 15 | 15 | 892.6 | 1,381 | 0.75 | 0.38–1.73 | indeterminate |
| Zidovudine | 25 | 23 | 1,418 | 1,745 | 1.59 | 0.98–2.85 | indeterminate |
| Atazanavir | 25 | 25 | 498.2 | 549.0 | 0.93 | 0.60–1.55 | indeterminate |
| Pegaspargase | 17 | 17 | 91 | 42 | 0.64 | 0.53–0.89 | early (alpha<1) |
| Nevirapine | 24 | 19 | 697.5 | 881.0 | 0.98 | 0.61–1.70 | indeterminate |
| Glutamine | 7 | 6 | 25 | 98 | 0.32 | 0.16–0.96 | early (alpha<1) |
| Indinavir | 16 | 16 | 364.8 | 577.5 | 0.79 | 0.45–1.73 | indeterminate |
| Darunavir | 14 | 13 | 432.2 | 430.0 | 0.72 | 0.41–1.31 | indeterminate |
| Tenofovir | 9 | 8 | 301.7 | 518.0 | 0.66 | 0.30–2.50 | indeterminate |
| Stavudine | 16 | 14 | 884.5 | 1,369 | 1.19 | 0.83–2.56 | indeterminate |
| Adefovir | 2 | n.a. | n.a. | n.a. | n.a. | n.a. | insufficient |
| Interferon alfa-2b | 13 | 12 | 79 | 63 | 0.72 | 0.44–1.35 | indeterminate |
| Arsenic Trioxide | 2 | n.a. | n.a. | n.a. | n.a. | n.a. | insufficient |
| Mercaptopurine | 17 | 17 | 267.3 | 488.0 | 0.81 | 0.46–1.90 | indeterminate |
| Nelfinavir | 22 | 17 | 458.4 | 537.0 | 0.78 | 0.43–1.44 | indeterminate |
| Satralizumab | 6 | 6 | 73 | 94 | 0.57 | 0.22–1.87 | indeterminate |
| Taliglucerase Alfa | 3 | n.a. | n.a. | n.a. | n.a. | n.a. | insufficient |
| Eliglustat | 2 | n.a. | n.a. | n.a. | n.a. | n.a. | insufficient |
| Mitoxantrone | 2 | n.a. | n.a. | n.a. | n.a. | n.a. | insufficient |

**Supplementary Figures**

**Supplementary Figure S1.** Two-directional sensitivity of the intermediate survivor set to the jaw-propensity threshold. (a) Number of drugs in the intermediate set when the jaw-contamination threshold is moved from 0.30 to 0.70 in steps of 0.05 (dashed line, primary threshold 0.50); annotations give the drugs that leave (−) or enter (+) the set relative to the primary set. (b) JPI of every four-criterion signal (ranked), with the swept band shaded; diamonds mark the final core (six glucocorticoids, asparaginase, pegaspargase), all of which lie below 0.30, and drugs inside the band are labelled. JPI = jaw-propensity index; GC = glucocorticoid.

**Supplementary Figure S2.** Adversarial glucocorticoid-misclassification tipping fractions for the 36 evaluable drugs. Bar length is the share of a drug's GC-negative osteonecrosis cases that would have to be reassigned to the GC-exposed stratum before the lower 95% bound of its GC-negative ROR reaches 1; labels give the corresponding number of reports and the size of the GC-negative stratum. Colours mark the fragile (< 0.30), moderate (0.30–0.59) and robust (≥ 0.60) bands; bars reaching 1.0 are right-censored at the 0.99 grid limit. GC = glucocorticoid; ROR = reporting odds ratio.

**Supplementary Figure S3.** Unmasking sensitivity analysis. (a) Natural-log ROR of the 34 negative controls under the primary universe, after removing every report with an antiresorptive or litigated antiretroviral as suspect drug (stage 1), and after additionally removing reports with a systemic glucocorticoid as suspect drug (stage 2); the dotted line marks ROR = 1 and the text gives the median ROR at each stage. (b) ROR of the 27 intermediate-set drugs under the primary universe versus the stage-1 unmasked universe (dashed line, identity); the drugs whose position changes most are labelled. ROR = reporting odds ratio.
